# DNA methylation biomarkers for early detection of ovarian cancer

**DOI:** 10.1007/s11033-026-12750-6

**Published:** 2026-09-11

**Authors:** Jeremy Yuan-Sheng Leow, Nancy Choon-Si Ng, Rebecca Shin-Yee Wong, Long Chiau Ming, Toong Lam Yap, Bey Hing Goh

**Affiliations:** 1 Department of Biomedical Sciences, Jeffrey Cheah Sunway Medical School, Faculty of Medical and Life Sciences, Sunway University, Sunway City, Malaysia; 2 Department of Medical Education, Jeffrey Cheah Sunway Medical School, Faculty of Medical and Life Sciences, Sunway University, Sunway City, Malaysia; 3 Graduate School of Medicine, KPJ Healthcare University, Nilai, Malaysia; 4 Faculty of Medicine, University of Cyberjaya, Cyberjaya, Malaysia; 5 Centre for Learning and Teaching, University of Cyberjaya, Cyberjaya, Malaysia; 6 School of Pharmacy, Faculty of Medical and Life Sciences, Sunway University, Sunway City, Malaysia; 7 Faculty of Health, Australian Research Centre in Complementary and Integrative Medicine, University of Technology Sydney, Ultimo, NSW Australia; 8 Sunway Biofunctional Molecules Discovery Centre, Faculty of Medical and Life Sciences, Sunway University, Sunway City, Malaysia; 9 Graduate Institute of Cancer Biology and Drug Discovery, College of Medical Science and Technology, Taipei Medical University, Taipei, Taiwan

**Keywords:** Cell-free DNA, DNA methylation, Early detection, Ovarian cancer, Screening

## Abstract

Ovarian cancer (OC) remains difficult to detect at an early stage, and current screening approaches using CA125 and transvaginal ultrasonography have not demonstrated sufficient benefit for population screening. DNA methylation is a promising biomarker class because epigenetic alterations may arise early in tumourigenesis, can be detected in circulating cell-free DNA (cfDNA), and may provide tissue-of-origin information. This review critically evaluates recent evidence on DNA methylation biomarkers for early OC detection. PubMed/MEDLINE, Web of Science, and Scopus were searched for studies published between January 2020 and September 2025, supplemented by selected earlier studies of biological or methodological relevance. Evidence was synthesised across single-gene biomarkers, multi-locus panels, genome-wide signatures, assay platforms, and machine-learning classifiers, with emphasis on early-stage performance, histological representation, comparator populations, analytical methodology, and validation design. Single-gene markers such as BRCA1, RASSF1A, OPCML, HOXA9, and HIC1 show variable performance, while multi-gene and classifier-based approaches generally provide stronger discrimination. However, many studies remain limited by retrospective case–control designs, small FIGO stage I–II subsets, predominance of serous disease, and insufficient prospective validation. Integration with CA125 may improve sensitivity but can reduce specificity, which is critical in low-prevalence screening. Clinical translation will therefore require minimal and reproducible methylation signatures, standardised low-input cfDNA workflows, rigorous external validation, and prospective longitudinal evaluation in intended-use populations.

## Introduction

Ovarian cancer (OC) remains an important cause of cancer morbidity and mortality among women worldwide. Although OC comprises a heterogeneous group of malignancies with distinct molecular and histological origins, accumulating pathological and molecular evidence supports the distal fallopian tube, particularly the fimbrial epithelium, as an important site of origin for a substantial proportion of high-grade serous ovarian carcinomas (HGSCs) [1–3]. According to GLOBOCAN 2022, OC accounted for 324,398 new cases and 206,839 deaths globally, illustrating its disproportionately high mortality relative to incidence [4]. This burden is driven partly by the high proportion of patients diagnosed after extra-pelvic dissemination, whereas outcomes are substantially more favourable when disease is detected at an early stage [5–8]. Large screening efforts combining serum CA125 with transvaginal ultrasonography have not demonstrated sufficient improvement in survival to support population screening, while CA125 itself has limited sensitivity for early-stage disease and may be elevated in benign and non-ovarian malignant conditions [7, 9–11]. These limitations are particularly consequential in a low-prevalence screening setting, where even modest false-positive rates can generate large numbers of unnecessary diagnostic investigations. It has been estimated that an effective early-stage OC screening test would require a sensitivity greater than 75% together with specificity of at least 99.6% to achieve a positive predictive value of 10% or greater [8].

Against this clinical background, aberrant DNA methylation represents an attractive biomarker class because epigenetic alterations may arise early during tumourigenesis, can influence transcriptional regulation without altering the DNA sequence, and can be detected in circulating tumour-derived DNA within the broader pool of cell-free DNA (cfDNA) [7, 12–14]. Cancer-associated methylation encompasses both locus-specific hypermethylation and broader alterations in methylation patterns, providing a potentially rich source of molecular information for cancer detection [1, 13, 14]. Importantly, methylation profiles can also contain information relevant to tissue of origin, which may help distinguish the anatomical source of a circulating cancer signal. This property is potentially valuable for OC detection because methylation alterations are not necessarily unique to ovarian malignancies and may overlap across different cancer types. Multi-cancer studies therefore provide important proof-of-concept for simultaneous cancer detection and tissue-of-origin localisation, but their performance should not be assumed to represent OC-specific diagnostic accuracy [15]. In addition, OC itself is epigenetically heterogeneous: methylation patterns derived predominantly from HGSC may not adequately represent endometrioid, clear-cell, mucinous, or low-grade serous tumours. Histotype composition must therefore be considered when evaluating candidate biomarkers and their generalisability.

Large-scale multi-cancer investigations illustrate both the potential and the limitations of methylation-based cfDNA detection. In the Circulating Cell-Free Genome Atlas (CCGA) case-control analysis, Liu et al. (2020) evaluated 6,689 participants and reported a validation specificity of 99.3% for a targeted cfDNA methylation classifier, together with tissue-of-origin localisation in patients with a cancer-like signal [15]. Within a prespecified group of 12 high-signal cancers that included OC, aggregate sensitivity increased from 39% for stage I disease to 69%, 83%, and 92% for stages II, III, and IV, respectively [15]. Importantly, these stage-stratified estimates were calculated across the prespecified cancer group rather than for OC alone and should therefore not be interpreted as stage-specific sensitivity estimates for OC. The marked stage dependence nevertheless highlights a central challenge for early detection: diagnostic performance obtained from cohorts containing substantial numbers of advanced-stage cancers may overstate the ability of an assay to identify clinically occult early-stage disease. This concern is also evident in the OC-specific literature, in which case numbers have generally been small and study populations have frequently been enriched for stage III–IV disease [7]. Accordingly, evaluation of methylation biomarkers for early detection should prioritise stage I–II performance, asymptomatic or intended-use populations, and clinically relevant comparator groups rather than relying on overall sensitivity or area under the curve derived from conventional retrospective case-control cohorts.

The field has consequently moved beyond isolated promoter-methylation events toward multi-locus signatures, genome-wide discovery approaches, and machine-learning classifiers. Plasma-based methylated DNA-marker panels have demonstrated the feasibility of detecting OC-associated methylation signals, including in early-stage disease, although early-stage subsets have often been small [2]. For example, Marinelli et al. identified an 11-marker plasma panel that detected all five early-stage HGSC cases included in their feasibility cohort, but the small number of early-stage cases limits the precision and generalisability of that estimate. More recently, large-scale cfDNA studies incorporating machine learning have reported encouraging early-stage discrimination and have explored integration with established biomarkers such as CA125 [16]. In one large cfDNA study, the addition of CA125 increased early-stage sensitivity but reduced specificity, illustrating that multimarker integration involves clinically important trade-offs rather than uniformly improving performance [16]. Other machine-learning studies have demonstrated highly accurate methylation-based classification in tissue; however, such findings cannot be directly extrapolated to blood-based screening. Gonzalez Bosquet et al. (2025), for example, developed a nine-probe HGSC classifier with strong internal and independent-dataset performance, but the study used surgical tissue samples and included only four stage I–II cases in its principal cohort [17]. These distinctions emphasise the need to separate biomarker discovery, retrospective diagnostic validation, external validation, and true intended-use screening evidence when assessing translational readiness.

Accordingly, this review critically evaluates DNA methylation biomarkers for early OC detection, with particular emphasis on evidence published between 2020 and 2025. Rather than considering reported diagnostic accuracy in isolation, we examine single-gene markers, multi-gene and differentially methylated region panels, genome-wide signatures, and classifier-based approaches in relation to cohort size, stage distribution, histological subtype, sample type, assay platform, comparator population, validation design, and performance specifically in early-stage disease. We further examine whether methylation-based approaches provide additive value beyond CA125, how benign ovarian and pelvic conditions and non-ovarian malignancies influence apparent specificity, and the extent to which tissue-of-origin approaches may address cross-cancer methylation overlap. Finally, we consider analytical constraints associated with low-input cfDNA, the risks of model overfitting and feature instability, and the level of prospective validation and clinical performance required before methylation-based assays could reasonably be considered for population-level early detection.

## Methods

### Literature search strategy

A structured literature search was conducted to identify studies evaluating DNA methylation as a biomarker for OC detection, with particular emphasis on early-stage disease and clinical translation. PubMed/MEDLINE, Web of Science, and Scopus were searched for peer-reviewed articles published between January 2020 and September 2025. This interval was selected to capture recent developments in circulating cell-free DNA (cfDNA) analysis, methylome-wide profiling, multiplex methylation assays, and data-driven classifier approaches. Earlier studies were retained selectively when they provided important methodological or biological context, or when more recent studies directly built upon their findings.

Search terms were combined using Boolean operators and covered four principal concepts: ovarian cancer, DNA methylation, circulating nucleic acids, and early detection. Terms included *“ovarian cancer”*, *“epithelial ovarian cancer”*, *“DNA methylation”*, *“methylome”*, *“hypermethylation”*, *“hypomethylation”*, *“differentially methylated region”*, *“cell-free DNA”*, *“cfDNA”*, *“circulating tumour DNA”*, *“ctDNA”*, *“plasma”*, *“serum”*, *“liquid biopsy”*, *“early detection”*, *“screening”*, *“diagnosis”*, *“biomarker”*, *“classifier”*, and *“machine learning”*. Search terms were adapted to the syntax of each database. Reference lists of relevant reviews and eligible primary studies were also manually screened to identify additional studies not retrieved by the electronic searches.

Where feasible, the full database-specific search strategies should be provided as supplementary material to improve reproducibility.

### Study selection and eligibility criteria

Primary human studies were considered eligible when they evaluated DNA methylation alterations with potential relevance to the detection or diagnosis of OC. Studies using plasma, serum, other blood-derived cfDNA or ctDNA, tumour tissue, or matched non-malignant tissue were considered where they contributed to biomarker discovery, analytical validation, or clinical evaluation. Particular emphasis was placed on studies involving early-stage disease (FIGO stages I–II), reporting stage-stratified results, or evaluating samples obtained from populations resembling the intended clinical-use setting.

Eligible studies included investigations of single-gene methylation markers, multi-gene panels, differentially methylated regions, genome-wide methylation signatures, and methylation-based computational or machine-learning classifiers. Studies were retained irrespective of whether they represented biomarker discovery, retrospective case-control validation, external validation, or prospective/intended-use evaluation; however, these study designs were distinguished during evidence synthesis because their reported diagnostic performance is not directly comparable.

Comparator populations were recorded explicitly and included healthy individuals, patients with benign ovarian or pelvic disease, and, where available, patients with non-ovarian malignancies. Studies comparing methylation-based approaches with established biomarkers such as CA125, or evaluating their combined performance, were specifically identified because of their clinical relevance.

Studies were excluded if they were confined to non-human models, investigated methylation solely for prognosis, treatment response, or therapeutic targeting without relevance to cancer detection, or described methylation alterations without evaluating potential diagnostic utility. Conference abstracts, editorials, commentaries, and non-peer-reviewed reports were excluded. Review articles were used to provide background information and to identify potentially relevant primary studies but were not treated as primary diagnostic evidence.

Where multiple publications originated from overlapping study populations, they were considered together and non-duplicative findings were extracted. When identical outcomes were reported more than once, greater weight was given to the report containing the larger cohort, more complete stage-specific data, or independent validation.

### Data extraction and evidence synthesis

For each eligible primary study, information was extracted on study design and setting, cohort size, comparator population, OC stage distribution, histological subtype, sample type, biomarker or methylation signature investigated, assay platform and methylation-detection chemistry, and whether the study included independent or external validation. For classifier-based studies, the number or type of methylation features, feature-selection approach, training and validation strategy, and evidence of external validation were also recorded where reported.

Diagnostic performance was extracted using the values reported by the original studies, including sensitivity, specificity, area under the receiver-operating-characteristic curve (AUC), positive predictive value (PPV), and negative predictive value (NPV), where available. Overall performance and performance specifically for FIGO stage I–II disease were recorded separately whenever possible. Comparisons with CA125 and changes in performance following combination of methylation markers with CA125 were also extracted when reported.

Comparator composition was considered when interpreting diagnostic performance because discrimination between OC and healthy controls may not reflect performance in clinically relevant populations containing benign ovarian or pelvic disease. Similarly, results from discovery and retrospective case-control cohorts were distinguished from those obtained in external validation, prospective, or intended-use populations.

Owing to substantial heterogeneity in study populations, methylation targets, analytical platforms, diagnostic thresholds, comparator groups, and reporting of performance outcomes, quantitative meta-analysis was not undertaken. Evidence was synthesised qualitatively, with emphasis on early-stage performance, reproducibility across independent cohorts, histological representation, analytical robustness, comparison with established biomarkers, and relevance to the intended clinical-use setting.

## Biological rationale for DNA methylation as an early detection signal in ovarian cancer

DNA methylation represents a biologically plausible biomarker class for early OC detection because aberrant methylation can arise during early tumourigenesis and can be detected in circulating tumour-derived DNA. Promoter hypermethylation is an early event in malignant transformation and may alter the expression of genes involved in tumour suppression, DNA repair, cell-cycle regulation, differentiation, and apoptosis [7, 13, 14, 18]. In parallel, broader hypomethylation across other genomic regions may contribute to genomic instability and aberrant activation of oncogenic pathways [1, 18]. Because these epigenetic alterations may be present during relatively early phases of tumour development, they provide a potential molecular signal for detecting disease before the clinical manifestations characteristic of advanced OC become apparent.

Importantly, OC is not a single epigenetic entity but comprises biologically distinct histological subtypes with different putative cells of origin and molecular characteristics. HGSC is strongly associated with precursor lesions arising in the distal fallopian tube, whereas endometrioid and clear-cell ovarian carcinomas are linked more closely to endometriosis; mucinous and low-grade serous tumours follow distinct developmental pathways [1, 18]. These biological differences extend to the epigenome. Although comparative methylation studies across OC histotypes remain limited, genome-wide analyses have reported greater overall DNA hypermethylation in HGSC than in lower-grade epithelial ovarian tumours, supporting the presence of subtype-associated methylation landscapes [1]. This heterogeneity has direct implications for biomarker development: methylation signatures discovered predominantly in HGSC cohorts may preferentially reflect HGSC biology and cannot be assumed to detect endometrioid, clear-cell, mucinous, or low-grade serous disease with equivalent sensitivity. Histotype composition should therefore be considered when interpreting both biomarker discovery and diagnostic performance.

At the molecular level, DNA methylation is mediated principally by DNA methyltransferases, which catalyse addition of a methyl group to cytosine residues, most commonly within CpG dinucleotides [12–14]. Cancer-associated methylation changes occur in different genomic contexts and may have different functional consequences. Hypermethylation of promoter-associated CpG islands can reduce transcription of tumour-suppressor and DNA-repair genes, whereas hypomethylation of promoters, enhancers, repetitive elements, or other genomic regions can contribute to aberrant gene expression and genomic instability [1, 13, 18]. In OC, recurrent methylation abnormalities have been described across tumour-suppressor genes, oncogenes, and regulatory pathways, indicating broad epigenetic reprogramming rather than alteration of a single methylation locus [1]. Consequently, the biological diversity of these alterations provides a rationale for moving beyond individual promoter markers toward multi-locus and methylome-wide signatures.

For blood-based detection, however, an important distinction must be made between cfDNA and circulating tumour DNA (ctDNA). Plasma cfDNA represents a heterogeneous mixture of DNA fragments released from multiple tissues through physiological and pathological cell turnover. In patients with cancer, a proportion originates from tumour cells through processes including apoptosis and necrosis, whereas substantial background cfDNA may arise from non-malignant sources such as leukocytes and bone-marrow-derived cells [5]. Thus, methylation profiles measured in total plasma cfDNA should not be interpreted as exclusively tumour-derived. Pre-analytical factors that increase cellular DNA contamination can further dilute the tumour-associated methylation signal; for example, delayed processing and inadequate plasma separation may increase high-molecular-weight DNA contamination [7]. This distinction is particularly relevant to early-stage detection, where the absolute amount of tumour-derived material available for analysis may be limited.

Methylation patterns nevertheless offer an additional advantage over many isolated genomic alterations because combinations of methylated regions can contain information relevant to the tissue from which the circulating DNA originated. Multi-cancer studies have demonstrated that methylation-based classifiers can simultaneously detect a cancer-associated signal and infer its likely tissue of origin; in the CCGA study, tissue-of-origin localisation was correct in 93% of samples for which a cancer-like signal and tissue prediction were generated [15]. However, tissue-of-origin inference should not be equated with complete cancer specificity. Individual methylation alterations may occur across several malignancies, and non-malignant tissues also contribute methylated DNA to the circulating cfDNA pool. Tissue-of-origin classification is therefore better regarded as a strategy for resolving overlapping methylation signals through multivariate patterns rather than evidence that a single methylated locus is inherently specific to OC.

The biological appeal of methylation-based liquid biopsy consequently derives from the combination of early epigenetic alteration, molecular diversity, detectability in circulating DNA, and the potential to capture tissue-related information. At the same time, these advantages are tempered by histological heterogeneity, background cfDNA from non-malignant tissues, overlap of methylation signals across cancers, and variation in circulating tumour-derived DNA abundance. These considerations support the development of carefully selected multi-locus signatures but also highlight why performance observed in tumour tissue or advanced-stage case-control cohorts cannot be assumed to translate directly into reliable early-stage OC screening.

## Single-gene, multi-gene, and classifier-derived DNA methylation biomarkers

The development of DNA methylation biomarkers for OC has progressed from targeted investigation of individual candidate genes toward multi-locus signatures and increasingly complex computational classifiers. These approaches differ not only in the number of methylation features interrogated but also in their stage of biomarker development, analytical requirements, and degree of clinical validation. Single-gene studies have provided important biological and diagnostic proof-of-concept but frequently show variable sensitivity across cohorts, whereas multi-gene panels aim to capture complementary epigenetic alterations and improve diagnostic discrimination. More recently, genome-wide discovery and machine-learning approaches have enabled the integration of larger numbers of methylation features into predictive models. The following sections critically examine these approaches with particular attention to early-stage performance, cohort composition, assay methodology, comparator populations, and the extent of independent or intended-use validation.

### Single-gene methylation biomarkers

Early investigations of DNA methylation in OC identified a wide range of candidate loci involving tumour-suppressor genes, developmental regulators, transcription factors, and signalling pathways. A systematic review of blood-derived methylation studies identified approximately 60 investigated genes, with RASSF1A, BRCA1, OPCML, APC, HIC1, and HOXA9 among the most frequently examined gene-specific targets [7]. Rather than implying that a small number of genes comprehensively represents the methylation landscape of OC, the following discussion focuses on recurrently investigated markers for which diagnostic performance has been reported across more than one study. Their performance nevertheless varies considerably according to biological specimen, assay design, comparator group, disease stage, and methylation threshold, limiting direct cross-study comparison.

BRCA1 is biologically relevant to OC because promoter hypermethylation can epigenetically silence BRCA1 and contribute to homologous recombination deficiency. However, its value as a diagnostic biomarker should be distinguished from its better-established investigation as a molecular, prognostic, and treatment-response marker. In an individual-participant meta-analysis of 2,636 patients from 15 studies, BRCA1 promoter methylation was detected in 430 tumours (16.3%) and was associated with younger age, advanced-stage disease, and high-grade serous histology; importantly, this analysis was designed to characterise clinicopathological and survival associations rather than early-detection performance [19]. The reported prevalence of BRCA1 methylation also varied markedly between studies, partly reflecting differences in assay methodology and methylation thresholds [19, 20]. In blood-based diagnostic studies summarised by Terp et al. (2023), single-marker sensitivity ranged from approximately 12% to 94%, while specificity ranged from approximately 49% to 100%, demonstrating substantial inter-study variability rather than consistently high discrimination [7]. Taken together, BRCA1 methylation remains biologically important in OC but currently lacks sufficiently consistent early-stage diagnostic performance to support its use as a stand-alone screening marker.

RASSF1A, a tumour-suppressor gene involved in cell-cycle control and apoptotic signalling, is one of the most extensively investigated methylation targets in OC. Nevertheless, its diagnostic performance is also heterogeneous. Across three studies in which single-marker blood-based performance could be evaluated, Terp et al. (2023) reported sensitivities of 25.4%, 37.7%, and 85.7%, with reported specificities ranging from 92.6% to 100% in studies providing specificity estimates [7]. These wide differences occurred even between studies employing broadly similar quantitative methylation-specific PCR approaches, suggesting that CpG target selection, assay design, sample characteristics, and threshold definition substantially influence apparent performance. Singh et al. (2021) similarly found RASSF1A methylation in 70% of stage I–II tumour samples, but this stage-specific observation was derived from tissue, not circulating cfDNA, and therefore should not be interpreted as 70% sensitivity for blood-based early detection [21]. Thus, although RASSF1A remains a biologically plausible and frequently investigated candidate, current evidence does not demonstrate sufficiently reproducible single-marker performance for population screening.

OPCML has shown comparatively encouraging results among recurrently studied single-gene methylation markers. OPCML is a tumour-suppressor gene that is frequently silenced through promoter methylation in OC. In the systematic evaluation by Terp et al. (2023), OPCML showed the strongest overall performance among the commonly investigated single-gene markers, with reported sensitivities across studies of approximately 80.0% to 97.8%; the highest reported estimate was accompanied by a specificity of 91.9% [7]. However, these findings have not been adequately reproduced in large independent prospective cohorts, and specificity in the low-90% range would remain insufficient for general-population OC screening, where false-positive rates must be extremely low. OPCML therefore illustrates an important distinction between promising case-control discrimination and evidence of clinical screening utility.

The developmental regulator HOXA9 and transcriptional repressor HIC1 have attracted particular interest because their methylation can be detected in circulating DNA. In the North Indian case-control study by Singh et al. (2021), promoter methylation of seven candidate genes was initially assessed in tissue samples from 85 EOC cases and 35 controls, including 20 stage I–II cancers; the three strongest-performing candidates, HOXA9, HIC1, and SOX1, were subsequently evaluated in serum cfDNA from 45 patients with EOC and 25 healthy controls [21]. In serum, HOXA9 showed 62.2% sensitivity, 100% specificity, and an AUC of 0.81, whereas HIC1 showed 71.1% sensitivity, 100% specificity, and an AUC of 0.88; SOX1 had lower sensitivity of 53.3% at 96% specificity. Stage-stratified analysis was encouraging but based on very small numbers: among only 10 stage I–II serum cases, HOXA9, HIC1, and SOX1 methylation were each detected in seven cases. These results demonstrate feasibility but should not be interpreted as precise estimates of early-stage sensitivity because of the small early-stage subset, retrospective case-control design, and use of healthy rather than clinically relevant benign controls.

Across the wider literature, HOXA9 has shown relatively consistent but modest single-marker sensitivity. Terp et al. (2023) reported sensitivities of approximately 59.5–62.2% across three analyses, with specificities of 95.3–100%. HIC1 showed sensitivities of approximately 71.1–78.8%, although specificity varied substantially, from 48.5% to 100% [7]. Therefore, although HOXA9 and HIC1 may offer greater consistency than some earlier single-gene markers, neither currently provides the combination of sensitivity and specificity required for population-level early detection when used alone.

Overall, the single-gene literature demonstrates that no individual methylation locus has yet shown sufficiently robust, externally validated, and stage-specific performance for standalone early OC screening. BRCA1 and RASSF1A illustrate substantial assay- and cohort-dependent variability, while OPCML, HOXA9, and HIC1 show more encouraging discrimination in selected studies but remain limited by retrospective designs, modest sample sizes, incomplete representation of early-stage and non-serous disease, and insufficient validation against benign or clinically relevant comparator populations. These limitations provide a biological and analytical rationale for combining complementary methylation signals into multi-gene panels rather than relying on a single epigenetic alteration.

### Emerging and less frequently investigated methylation biomarkers

Beyond recurrently investigated loci such as RASSF1A, BRCA1, OPCML, HOXA9, and HIC1, a substantially broader range of methylation alterations has been explored as potential OC biomarkers. These candidates include tumour-suppressor genes, developmental regulators, signalling-associated genes, and differentially methylated regions identified through genome-wide profiling. Their inclusion is important because restricting biomarker evaluation to historically well-characterised genes may overlook methylation alterations with greater sensitivity, histotype specificity, or complementarity within multi-marker panels. However, most emerging candidates remain at the discovery or early analytical-validation stage, and evidence of differential methylation should not be equated with demonstrated diagnostic performance.

Candidate-gene studies have continued to broaden the range of potentially informative loci. Singh et al. (2021) evaluated seven hypermethylated genes—RASSF1A, DAPK1, SOX1, HOXA9, HIC1, SPARC, and SFRP1—using quantitative MethyLight assays in 85 epithelial ovarian cancer (EOC) tissues and 35 control tissues [21]. Methylation frequencies across the seven genes ranged from approximately 61% to 82%, with HOXA9, HIC1, and SOX1 showing the strongest single-marker performance and subsequently being prioritised for serum cfDNA analysis. Importantly, the remaining candidates—DAPK1, SFRP1, SPARC, and RASSF1A—were not all independently evaluated as circulating biomarkers in the serum cohort. Thus, their tumour-tissue methylation frequencies should be regarded primarily as evidence supporting candidate selection rather than evidence of blood-based early-detection performance. This distinction illustrates a recurring challenge in the field: many reported methylation markers progress from tissue discovery to nomination as potential biomarkers, but substantially fewer undergo independent validation in circulating DNA.

An additional emerging candidate is CYB5R4. Gonc et al. (2025) assessed CYB5R4 methylation in blood-derived DNA from 387 patients with serous OC, 50 individuals with benign ovarian disease, and 100 healthy controls [22]. Median methylation was higher in OC than in benign and healthy groups (6.23% versus 3.52% and 4.47%, respectively), although the absolute separation between groups was modest. The relatively large OC cohort and inclusion of benign ovarian comparators are strengths, particularly because benign masses represent a more clinically relevant differential-diagnosis group than healthy controls alone. However, the study did not establish conventional diagnostic performance measures such as sensitivity, specificity, or AUC for CYB5R4 as a screening test. Moreover, the methodological description indicates that DNA was isolated from lymphocyte samples, rather than specifically from plasma cfDNA, despite discussion of the findings in the context of circulating tumour DNA. Accordingly, CYB5R4 should presently be regarded as a blood-associated methylation candidate requiring further analytical clarification and validation rather than an established ctDNA biomarker.

More recent studies have shifted from predetermined candidate genes toward genome-wide methylation discovery. Zhou et al. (2025) employed TET-assisted pyridine borane sequencing (TAPS)-based whole-genome methylation profiling to compare plasma cfDNA from patients with EOC and benign pelvic masses [23]. The initial analysis identified widespread differential methylation and subsequently prioritised 35 differentially methylated genes, from which NBL1 and CASZ1 emerged as leading candidates. Integration with The Cancer Genome Atlas (TCGA) data, including 43 stage I–II OC tissues and 12 normal tissues, further narrowed the candidate regions, and targeted tissue validation demonstrated hypermethylation at selected sites in NBL1 and CASZ1. NBL1 showed the more biologically coherent pattern: increased methylation was accompanied by reduced mRNA expression and lower protein expression in cancer tissue, whereas CASZ1 showed discordant methylation patterns between blood and tissue.

Despite these findings, the evidence for NBL1 as an early-detection biomarker remains preliminary. The prospective plasma discovery component comprised only five EOC cases and five benign pelvic-mass controls, and only one of the five plasma OC cases was stage I–II; the remaining cases were advanced-stage disease. The separate tissue-validation cohort similarly comprised five EOC cases and five benign controls, with all five cancers being stage III–IV. Although the use of stage I–II TCGA samples strengthens biological validation, these were tissue data rather than independent early-stage plasma samples. Furthermore, the study did not report sensitivity, specificity, AUC, PPV, or NPV for NBL1 as a diagnostic assay. NBL1 should therefore be considered a promising discovery-stage methylation candidate, rather than a validated biomarker for early-stage OC screening.

Taken together, emerging methylation studies demonstrate two complementary directions in biomarker development. Candidate-gene approaches continue to identify additional loci that may contribute useful signals within multiplex assays, whereas genome-wide approaches enable less hypothesis-driven identification of novel differentially methylated regions. However, progression from differential methylation to a clinically useful biomarker requires more than biological plausibility. Candidate loci must demonstrate reproducible detection in blood-derived cfDNA, stage I–II sensitivity at appropriately high specificity, discrimination from benign ovarian and pelvic conditions, consistency across histological subtypes and populations, and independent prospective validation. At present, most less frequently investigated markers have not yet met these requirements, supporting their interpretation primarily as candidates for incorporation into future multi-locus signatures rather than as stand-alone screening biomarkers.

### Multi-gene methylation panels and integrated biomarker models

The heterogeneous methylation landscape of OC provides a rationale for combining multiple epigenetic targets rather than relying on a single locus. Multi-gene or multi-region assays may improve sensitivity by capturing complementary tumour-associated alterations, while requiring concordant or threshold-based methylation patterns may help preserve specificity. However, apparent gains in diagnostic performance should be interpreted in relation to cohort composition, stage distribution, comparator population, and validation strategy because most published studies remain retrospective or case–control in design.

A relatively simple example is provided by Singh et al. (2021), who evaluated combinations of HOXA9, HIC1, and SOX1 using multiplex MethyLight assays in serum cfDNA from 45 patients with EOC and 25 healthy controls [21]. Individually, HOXA9 and HIC1 showed sensitivities of 62.2% and 71.1%, respectively, whereas the HOXA9–HIC1 combination achieved 88.9% sensitivity, 100% specificity, and an AUC of 0.95. The HIC1–SOX1 combination achieved 80.0% sensitivity and 96% specificity, with an AUC of 0.93. These findings provide direct within-cohort evidence that combining methylation signals can improve discrimination relative to the constituent markers. Stage-specific results were also encouraging: both HOXA9–HIC1 and HIC1–SOX1 were positive in all 10 stage I–II serum cases. However, the early-stage subset comprised only 10 patients, controls were healthy individuals rather than women with benign pelvic disease, and the reported training/test assessment was based on random partitioning of the same study population rather than validation in a fully independent external cohort. These limitations substantially reduce the precision and generalisability of the reported early-stage performance.

A broader methylome-discovery approach was used by Marinelli et al. (2022). Reduced-representation bisulfite sequencing of OC tissue, benign fallopian-tube epithelium, and buffy-coat controls was followed by independent tissue validation and selection of 11 methylated DNA markers (GPRIN1, CDO1, SRC, SIM2, AGRN, FAIM2, CELF2, RIPPLY3, GYPC, CAPN2, and BCAT1) for plasma testing [2]. The plasma cohort comprised 91 women with OC and 91 cancer-free controls, with high-grade serous disease accounting for 73 of 91 cancers. A random-forest model incorporating the 11 markers achieved 79% sensitivity at 96% specificity, with an AUC of 0.91, following 500-fold in-silico cross-validation. All five stage I–II high-grade serous cancers included in the plasma cohort were identified. Although this result supports the feasibility of detecting early-stage methylation signals, the early-stage estimate is based on only five cases and therefore cannot establish precise stage I–II sensitivity. Moreover, the study did not evaluate pre-diagnostic screening samples, and its plasma performance was internally cross-validated rather than independently reproduced in an intended-use population.

The distinction between diagnostic case–control performance and true early-detection performance is illustrated particularly clearly by Herzog et al. (2024). The investigators developed the WID-cfOC score, based on methylation in three regions located within ZNF154, C2CD4D, and WNT6, using targeted bisulfite sequencing. In a diagnostic cohort comprising 27 °C cases, four healthy volunteers, and 37 women with benign pelvic pathologies, the assay achieved 97.6% specificity and 66.7% sensitivity for all cancers, increasing to 80% sensitivity for grade 2–3 cancers [24]. The inclusion of women with benign pelvic abnormalities is clinically important because it provides a more demanding comparator population than healthy controls alone.

Herzog et al. (2024) also directly evaluated whether methylation provided additive value to CA125. Among high-risk cancers in the diagnostic cohort, WID-cfOC showed 77.8% sensitivity and 97.4% specificity, compared with 83.3% sensitivity and 87.2% specificity for CA125. Defining a positive combined test as positivity for either methylation or CA125 increased sensitivity to 94.4%, but specificity fell to 87.2% [24]. Thus, addition of CA125 did not produce an unqualified improvement: greater sensitivity was obtained at the expense of substantially more false-positive results. This trade-off is particularly important in OC screening, where specificity is critical because of the low disease prevalence and potential harms of invasive follow-up.

More importantly, performance was markedly lower when the same methylation score was applied to samples obtained before clinical diagnosis. The early-detection cohort was derived from the UK Familial Ovarian Cancer Screening Study and included 29 women who subsequently developed OC and 29 risk-matched controls. Although specificity remained 100%, sensitivity for high-risk cancers with relatively lower genomic-DNA contamination was only 27.3%, increasing to 33.3% in samples obtained within one year of diagnosis. In the subset with contemporaneous CA125 data, cfDNA methylation showed 22.2% sensitivity, compared with 44.4% for CA125, and combining the two tests did not improve sensitivity beyond CA125 alone. Stage-specific analysis further demonstrated the challenge: neither methylation nor CA125 detected any stage I cancers in the diagnostic cohort, while only one stage II cancer was identified; in the pre-diagnostic cohort, one of two evaluable stage II cancers was detected. By contrast, most stage III–IV cases in the diagnostic cohort were identified. Although archival sample quality and genomic-DNA contamination probably contributed to the lower pre-diagnostic sensitivity, these findings illustrate why diagnostic case–control performance cannot be extrapolated directly to screening performance.

Collectively, these studies support the biological premise that integrating multiple methylation signals can improve discrimination over some constituent single markers, but the magnitude and clinical relevance of this improvement remain context dependent. Small early-stage subsets can generate apparently high stage I–II sensitivity, while performance may fall substantially when assays are applied to pre-diagnostic samples. Similarly, combining methylation markers with CA125 can increase sensitivity in some diagnostic cohorts but may simultaneously reduce specificity, and additive benefit has not been demonstrated consistently in pre-diagnostic settings. For average-risk population screening, these trade-offs are particularly important because modelling suggests that sensitivity of at least 75% would need to be accompanied by specificity of approximately 99.6% to attain an acceptable positive predictive value [25].

Accordingly, the principal translational question is no longer whether multi-locus methylation panels can distinguish established OC from controls, but whether a stable and analytically tractable signature can detect stage I–II disease at very high specificity in independent, prospectively collected intended-use populations. Such validation should include adequate numbers of early-stage cases, benign ovarian and pelvic conditions, representation of non-serous histotypes, and direct comparison with—or assessment of incremental value beyond—established biomarkers such as CA125.

### Machine learning and methylation-based classifiers

Advances in computational modelling have enabled the analysis of high-dimensional methylation data in which diagnostic information may be distributed across large numbers of CpG sites rather than confined to a small set of predefined genes. Machine-learning approaches can integrate these correlated methylation features, perform dimensionality reduction, and derive multivariate decision functions for cancer classification. However, machine learning does not eliminate the need for careful biomarker selection or validation: model performance remains dependent on the characteristics of the training population, the number and stability of selected features, assay platform, class balance, and the extent to which model development is separated from independent testing.

A large cfDNA-based example was reported by G. Li et al. (2024). The investigators initially surveyed more than 3.3 million CpG sites using pooled cfDNA samples and subsequently evaluated 493 candidate methylation markers in an individual cohort comprising 754 patients with EOC, including 205 with early-stage disease, and 1,118 healthy women [16]. They pretrained a transformer-based methylation model, MethylBERT, using methylation data from more than 110,000 cancer samples and subsequently fine-tuned the model using the 493 selected cfDNA methylation features. The individual cohort was randomly divided into training and validation sets. In the training set of 503 EOC cases and 744 controls, MethylBERT-EOC achieved 93.24% sensitivity at 95.3% specificity (AUC 0.98); in the held-out validation set of 251 EOC cases and 374 controls, sensitivity was 89.24% at 94.39% specificity (AUC 0.97). Among early-stage cases, sensitivity decreased from 84.09% (111/132) in training to 79.45% (58/73) in validation, demonstrating the importance of reporting stage-specific rather than overall classifier performance [16].

The operating threshold of a classifier is particularly important when its intended application is screening. In the validation cohort of G. Li et al. (2024), increasing specificity from approximately 95% to 99.2% reduced early-stage sensitivity from 79.45% to 63.01%, while overall sensitivity fell to 70.92% [16]. This trade-off is clinically important because the specificity required for population-level OC screening is considerably higher than that commonly selected to maximise AUC or balanced classification accuracy. The same study also examined 96 independent endometriosis samples as a more clinically relevant comparator group; the model distinguished EOC from endometriosis with 89.24% sensitivity and 91.66% specificity, indicating that performance against healthy controls may not fully represent discrimination from benign gynaecological disease.

Direct comparison with a conventional statistical classifier further suggested that model architecture can influence performance. Using the same methylation dataset, G. Li et al. (2024) developed a LASSO-logistic regression model based on 21 selected markers. In the validation cohort, the LASSO model achieved 83.67% sensitivity at 89.04% specificity (AUC 0.92), compared with 89.24% sensitivity at 94.39% specificity for MethylBERT. For early-stage disease, LASSO identified 49 of 73 cases (67.12% sensitivity), approximately 12% points lower than MethylBERT in the same validation population. These within-cohort comparisons provide stronger evidence for improved classification than comparisons between models developed in unrelated populations. Nevertheless, the MethylBERT training and validation samples were generated by random partitioning of the same retrospective individual cohort; thus, the validation set represents an internal held-out assessment rather than fully independent external validation of the classifier.

Histological heterogeneity also remains relevant to classifier performance. In the MethylBERT validation dataset, early-stage sensitivity was 76.74% for serous carcinoma, 72.73% for endometrioid carcinoma, 87.5% for mucinous carcinoma, and 83.33% for clear-cell carcinoma; however, the corresponding early-stage subsets contained only 43, 11, eight, and six cases, respectively. These estimates therefore remain imprecise, particularly for non-serous histotypes, and require confirmation in substantially larger subtype-specific cohorts. Similarly, combining MethylBERT with CA125 increased sensitivity among 183 early-stage cases from 82.51% to 89.62%, but specificity decreased from 97.36% to 93.55%, again demonstrating that addition of a clinical biomarker may improve sensitivity at the cost of increased false-positive classification [16].

A complementary tissue-based proof-of-concept was reported by Gonzalez Bosquet et al. (2025). The study analysed surgical specimens from 99 patients with high-grade serous ovarian carcinoma (HGSC) and 12 normal fallopian-tube controls, using more than 850,000 methylation probes from the Infinium MethylationEPIC array [17]. Only four HGSC cases were stage I–II, while most were stage III–IV. A deep-learning feature-selection approach using MethylNet initially identified 23,397 informative probes with an AUC of 1.00 in the development dataset. Subsequent ANOVA and LASSO reduction yielded a simplified nine-probe signature, which also achieved an AUC of 1.00 during model development. Importantly, external validation demonstrated different behaviour: the larger 11,167-probe model retained an AUC of 0.98, whereas the simplified nine-probe model achieved a more modest AUC of 0.84 (95% CI 0.76–0.93) in an independent dataset containing 114 HGSC and seven fallopian-tube samples. This reduction in performance illustrates why very high apparent accuracy in a development cohort should not be assumed to persist after feature reduction or external validation.

The study by Gonzalez Bosquet et al. (2025) also illustrates several additional translational limitations. The authors acknowledged a potential “black-box” effect in the deep-learning feature-selection process and noted that the relatively homogeneous study population, although advantageous for model development, could restrict generalisability. The classifier was developed entirely from tumour and fallopian-tube tissue rather than plasma cfDNA, focused specifically on HGSC, and was evaluated predominantly in individuals of similar Western European ancestry. The authors therefore emphasised that translation to early detection would require validation using blood samples from diverse populations, representation of all disease stages, and comparator groups including women with benign pelvic masses [17]. Accordingly, its current value is primarily as proof-of-concept for methylation-feature discovery and dimensionality reduction rather than as evidence of screening-ready performance.

Taken together, these studies demonstrate the potential of machine learning to extract diagnostically informative patterns from high-dimensional methylation datasets, but they also illustrate several distinct levels of validation that should not be considered interchangeable. Internal cross-validation or random train–test splitting assesses model performance within a source population; external validation tests transportability to an independent cohort; and prospective evaluation in pre-diagnostic or intended-use samples is required to establish screening performance. High AUC values in tissue-based or retrospective case–control datasets may therefore overestimate clinical utility, particularly when early-stage cases are sparse, healthy individuals constitute the primary control group, or large numbers of candidate features are selected relative to the number of cases.

Future methylation-classifier development should consequently prioritise prespecified and locked feature sets, transparent reporting of feature-selection and model-training procedures, independent external validation, and evaluation across assay platforms, ethnic populations, histological subtypes, and clinically relevant benign comparator groups. For early-detection applications, performance should be reported specifically for FIGO stage I–II disease and at specificity thresholds appropriate to low-prevalence screening rather than solely at thresholds that maximise AUC or overall classification accuracy. Ultimately, prospective evaluation in asymptomatic or high-risk populations will be required to determine whether increasingly sophisticated computational models provide clinically meaningful improvement over simpler and more readily transferable methylation assays.

Table 1 summarises the principal gene-specific markers, multi-locus methylation panels, emerging biomarkers, and classifier-based approaches evaluated for OC detection, including study design, cohort composition, stage distribution, assay platform, validation strategy, and reported diagnostic performance.

**Table 1:** Representative recent DNA methylation biomarkers, panels, and classifiers evaluated for ovarian cancer detection

Study / approach	Biomarker / signature	Design / validation	Cohort, stage and histotype	Specimen / assay	Diagnostic performance	CA125 comparison / key limitation
**M. C. Liu et al. (2020)** CCGA multi-cancer classifier	> 100,000 informative methylation regions; multi-cancer detection and tissue-of-origin classifier	Prospective case-control sub-study; training and independent validation within CCGA	6,689 participants: 2,482 cancers (> 50 cancer types) and 4,207 non-cancer controls. Ovary included among 12 prespecified high-signal cancers.	Plasma cfDNA; targeted bisulfite sequencing	Validation specificity 99.3%. Across the 12 prespecified cancers, sensitivity: stage I 39%, II 69%, III 83%, IV 92%. Tissue-of-origin localization correct in 93% of samples with a cancer-like signal and predicted origin.	No OC-specific CA125 comparison. Stage-specific sensitivities are aggregate across 12 cancers, not ovarian-specific; proof-of-concept rather than OC screening validation.
**Singh et al. (2021)** Candidate genes and 2-gene panels	HOXA9, HIC1, SOX1; panels HOXA9 + HIC1, HIC1 + SOX1, HOXA9 + SOX1	Retrospective case-control; tissue candidate evaluation followed by serum analysis; internal data partitioning rather than independent external serum validation	Tissue: 85 EOC + 35 controls. Serum: 45 EOC + 25 healthy controls; stage I-II *n* = 10, III *n* = 30, IV *n* = 5; predominantly serous.	Serum cfDNA; bisulfite conversion; singleplex/multiplex MethyLight qMSP; selected tissue loci checked by clonal bisulfite sequencing	Single genes: HOXA9 62.2%/100% (AUC 0.81); HIC1 71.1%/100% (0.88); SOX1 53.3%/96% (0.77). Panels: HOXA9 + HIC1 88.9%/100% (0.95); HIC1 + SOX1 80.0%/96% (0.93); HOXA9 + SOX1 66.7%/96% (0.85). Stage I-II: 10/10 positive for HOXA9 + HIC1 and HIC1 + SOX1; 8/10 for HOXA9 + SOX1.	CA125 assessment was limited and did not provide a robust paired full-cohort sensitivity/specificity comparison. Very small early-stage subset and healthy controls limit generalisability.
**N. Li et al. (2022)** Blood-based methylation classifier	1,272 tissue-derived DMRs incorporated into a supervised machine-learning classifier	Tissue discovery; blood training with 5-fold cross-validation; independent blood test cohort	Tissue *n* = 152; blood *n* = 373. Training *n* = 178; independent test *n* = 184. Test malignant cases: stage I-II *n* = 9, III *n* = 59, IV *n* = 6, unknown *n* = 11; healthy *n* = 53; benign ovarian tumours *n* = 46.	Blood-derived DNA; targeted bisulfite sequencing	Training AUC 0.94. Independent test: non-malignancy correctly classified in 96.2% of healthy and 93.5% of benign samples; malignant detection 44.4% stage I-II, 86.4% stage III, 100% stage IV; overall accuracy 89.5%.	No direct CA125 performance comparison reported in the primary test results. Early-stage sensitivity was low and based on only 9 stage I-II cancers despite strong overall accuracy.
**Marinelli et al. (2022)** 11-MDM plasma panel	GPRIN1, CDO1, SRC, SIM2, AGRN, FAIM2, CELF2, RIPPLY3, GYPC, CAPN2, BCAT1	RRBS discovery; independent tissue validation; plasma feasibility cohort with 500-fold in-silico cross-validation	Plasma: 91 °C + 91 cancer-free controls; 73/91 (80%) high-grade serous. Stage I-II HGSOC *n* = 5.	Discovery by RRBS; independent tissue MSP; plasma TELQAS assay; random-forest model	Sensitivity 79% (95% CI 69–87), specificity 96% (89–99), AUC 0.91 (0.86–0.96). All 5 stage I-II HGSOC cases detected.	No direct CA125 comparison. Early-stage estimate based on only 5 cases; plasma performance internally cross-validated and not tested in pre-diagnostic/intended-use samples.
**Herzog et al. (2024)** WID-cfOC	Three regions within ZNF154, C2CD4D and WNT6	Diagnostic case-control cohort plus independent pre-diagnostic UKFOCSS samples	Diagnostic: 27 °C + 41 controls (4 healthy, 37 benign pelvic); known stages I *n* = 3, II *n* = 1, III *n* = 10, IV *n* = 7; 19 HGSC. Pre-diagnostic: 29 future OC + 29 matched controls; stages I *n* = 7, II *n* = 2, III *n* = 15, IV *n* = 2 among cases.	Plasma cfDNA; targeted bisulfite sequencing	Diagnostic: 66.7% sensitivity, 97.6% specificity overall; 80% sensitivity for grade 2–3 cancers. Pre-diagnostic: specificity 100%; high-risk sensitivity 27.3% in lower-gDNA samples and 33.3% <1 year before diagnosis. Neither test detected stage I cancer in diagnostic set.	High-risk diagnostic subset: cfDNAme 77.8%/97.4%; CA125 83.3%/87.2%; OR-combination 94.4%/87.2%. In lower-gDNA pre-diagnostic subset: cfDNAme 22.2%, CA125 44.4%, combination 44.4% sensitivity, all at 100% specificity. Small cohorts and archival sample quality limit inference.
**G. Li et al. (2024)** MethylBERT-EOC	493 cfDNA methylation markers selected from > 3.3 million CpGs; transformer-based classifier	Retrospective individual cohort randomly split into training and held-out validation; not independent external classifier validation	Individual cohort: 754 EOC (205 early-stage) + 1,118 healthy women. Validation: 251 EOC + 374 healthy; stage I-II *n* = 73. Separate endometriosis comparator *n* = 96.	Plasma cfDNA; targeted methyl-capture sequencing; pretrained MethylBERT model	Validation: 89.24% sensitivity, 94.39% specificity, AUC 0.97; stage I-II 79.45% (58/73). At 99.2% specificity, stage I-II sensitivity 63.01%. EOC vs. endometriosis: 89.24% sensitivity, 91.66% specificity.	With CA125 (*n* = 1,058): overall sensitivity 92.47% to 95.68%, but specificity 97.36% to 93.55%; stage I-II sensitivity 82.51% to 89.62%. Sensitivity gain therefore accompanied by lower specificity; same-source train/validation split.
**G. Li et al. (2024)** OV1 ddPCR + prospective high-risk cohort	OV1 single methylation marker translated from sequencing discovery to targeted ddPCR	Separate ddPCR cohort with train/validation split; then prospective high-risk screening cohort using OV1 + CA125 as first-line tests	ddPCR cohort: 305 EOC + 480 healthy; early-stage *n* = 111, advanced *n* = 194. Prospective high-risk cohort: 2,117 women; positive first-line tests followed by TVU, MRI if suspicious, and surgery/histology when indicated.	Plasma cfDNA; bisulfite conversion + targeted ddPCR	ddPCR validation: 72.16% sensitivity, 92.95% specificity, AUC 0.877. Early-stage: 57.66% sensitivity at 92.71% specificity. Prospective: 314 first-line positives; 4 cancers confirmed among them (3 stage I, 1 stage III), plus 1 cancer among initially negative participants. Authors estimated sensitivity 80% and specificity 85.3%.	Early-stage CA125: 38.74% sensitivity at 95% specificity; OV1 + CA125: 72.07% sensitivity at 88.12% specificity. Prospective estimates depend on assumed disease status for many participants, short follow-up, and imaging rather than universal histologic confirmation.
**Gonzalez Bosquet et al. (2025)** Tissue AI classifier	MethylNet feature set (23,397 probes); ANOVA 11,167-probe model; simplified 9-probe LASSO signature	Tissue model development with cross-validation; independent external GEO validation	Development: 99 HGSC + 12 normal fallopian tubes; only 4 stage I-II, 62 stage III, 26 stage IV, 6 recurrent. External: 114 HGSC + 7 fallopian tubes.	Surgical tissue; Infinium MethylationEPIC array (> 850,000 probes); MethylNet, ANOVA and LASSO	Development AUC 1.00 for high-dimensional and 9-probe models. External validation: AUC 0.98 for 11,167-probe model; AUC 0.84 (95% CI 0.76–0.93) for 9-probe model.	No blood-based or CA125 diagnostic comparison. HGSC-only tissue study with very few early-stage cases and relatively homogeneous ancestry; illustrates feature-reduction/generalisation trade-off.
**Gonc et al. (2025)** CYB5R4	CYB5R4 methylation	Cross-sectional case-control biomarker association study	387 serous OC + 50 benign ovarian disease + 100 healthy controls. OC stages: I *n* = 61, II *n* = 42, III *n* = 204, IV *n* = 52, unknown *n* = 28.	Peripheral blood-derived DNA; methylation-sensitive restriction enzyme assay with qPCR quantification; 6% methylation threshold	Median methylation: OC 6.23%, benign 3.52%, healthy 4.47%; 229/387 °C samples classified as methylated. Conventional diagnostic sensitivity, specificity and AUC were not established.	No CA125 diagnostic comparison. Signal was more frequent in advanced-stage disease; not a plasma-cfDNA validation study and should be considered an emerging blood-associated marker.
**Zhou et al. (2025)** Genome-wide TAPS discovery	35 differentially methylated genes; NBL1 and CASZ1 prioritised	Prospective exploratory plasma discovery with tissue/TCGA biological validation	Overall study: 10 EOC + 10 benign pelvic masses. Plasma discovery subset 5 EOC + 5 benign; only 1 plasma EOC was stage I-II. Separate tissue validation 5 EOC + 5 benign (all EOC stage III-IV); TCGA included 43 stage I-II OC tissues + 12 normal tissues.	Plasma ctDNA; whole-genome TAPS; candidate validation by bisulfite sequencing, qRT-PCR and IHC	NBL1 hypermethylation showed concordance between blood/tissue and inverse association with expression; CASZ1 showed discordant blood/tissue patterns. No sensitivity, specificity, AUC, PPV or NPV reported.	No CA125 comparison. Discovery-stage study with very small plasma cohort and only one early-stage plasma case; not yet a clinically validated diagnostic assay.

Abbreviations: AUC, area under the receiver operating characteristic curve; CA125, cancer antigen 125; CCGA, Circulating Cell-Free Genome Atlas; cfDNA, cell-free DNA; ctDNA, circulating tumour DNA; ddPCR, droplet digital polymerase chain reaction; DMR, differentially methylated region; EOC, epithelial ovarian cancer; HGSOC/HGSC, high-grade serous ovarian cancer/carcinoma; IHC, immunohistochemistry; MRI, magnetic resonance imaging; MSP, methylation-specific polymerase chain reaction; MSRE, methylation-sensitive restriction enzyme; qMSP, quantitative methylation-specific polymerase chain reaction; qRT-PCR, quantitative reverse-transcription polymerase chain reaction; RRBS, reduced-representation bisulfite sequencing; TAPS, TET-assisted pyridine borane sequencing; TELQAS, Target Enrichment Long-probe Quantitative Amplified Signal; TVU, transvaginal ultrasonography; UKFOCSS, UK Familial Ovarian Cancer Screening Study

## Assay platforms and analytical considerations for DNA methylation profiling in ovarian cancer

The performance of a DNA methylation biomarker depends not only on the biological relevance of the selected locus or signature but also on the analytical platform used to measure it. Available approaches differ substantially in genomic coverage, quantitative resolution, DNA-input requirements, susceptibility to conversion or enrichment bias, sequencing burden, and potential for clinical scalability. These considerations are particularly important for early-stage OC, where tumour-derived DNA may constitute only a small component of circulating cell-free DNA and analytical loss or background signal can therefore materially affect detection. Current OC studies span targeted PCR-based assays, bisulfite sequencing, emerging bisulfite-free enzymatic methods, and enrichment-based methylome profiling. The following sections compare these approaches with emphasis on their suitability for low-input cfDNA analysis, major sources of analytical error, and current level of OC-specific validation.

### MSP, qMSP, and ddPCR (Targeted assays)

Targeted PCR-based methylation assays remain widely used because they allow sensitive interrogation of predefined loci without the sequencing depth and computational requirements associated with genome-wide profiling. These technologies are not specific to OC, but their relatively focused analytical design makes them attractive for translating methylation candidates discovered in tissue or genome-wide studies into assays suitable for blood-based testing. Methylation-specific PCR (MSP) provides primarily qualitative or semi-quantitative discrimination between methylated and unmethylated DNA, whereas quantitative MSP (qMSP), including MethyLight-based approaches, enables quantitative estimation of methylation using real-time amplification. qMSP is comparatively rapid and cost-effective but remains dependent on bisulfite conversion, methylation-specific primer design, and the CpG sites selected for interrogation [7, 13, 26].

These assay-design considerations are particularly relevant in OC because apparently similar PCR-based assays can produce substantially different biomarker performance. Terp et al. (2023) found that PCR-based methods were the most commonly used approaches in blood-derived OC methylation studies, including conventional MSP, nested MSP, qMSP, digital MSP, and methylation-specific ddPCR [7]. However, even studies using real-time qMSP reported markedly different sensitivities for the same gene when different CpG regions were targeted, indicating that assay design itself can influence apparent biomarker performance. Singh et al. (2021), for example, used singleplex and multiplex MethyLight assays to quantify HOXA9, HIC1, and SOX1 methylation in serum cfDNA and demonstrated concordance with clonal bisulfite sequencing [21]. The multiplex format also enabled simultaneous interrogation of multiple loci from limited template DNA, illustrating a practical advantage of targeted qMSP for low-input liquid-biopsy samples. Nevertheless, quantitative values and diagnostic thresholds remain assay-specific, meaning that cut-offs established in one cohort cannot be assumed to transfer directly across laboratories, primer sets, or populations.

Droplet digital PCR (ddPCR) provides an alternative targeted strategy in which DNA molecules are partitioned across large numbers of individual reactions, allowing digital counting of positive and negative partitions and more direct quantification of low-abundance targets. Compared with conventional qPCR-based methods, this can be advantageous when the methylated fraction is small, although ddPCR remains restricted to predefined loci and requires carefully designed primers and probes [26]. An OC-specific example was provided by G. Li et al. (2024), who transferred the methylation marker OV1, initially identified through large-scale cfDNA methylation profiling, to a targeted ddPCR assay. In an independent ddPCR cohort of 305 patients with EOC and 480 healthy women, OV1 achieved 72.16% sensitivity at 92.95% specificity in the validation dataset; among 111 early-stage cases, sensitivity was 57.66% at 92.71% specificity [16]. These findings demonstrate the potential value of ddPCR as a lower-complexity assay for translating sequencing-derived methylation markers, but they also show that high analytical sensitivity does not necessarily translate into sufficiently high clinical sensitivity for early-stage disease.

Importantly, the practical limit of detection of any targeted methylation assay is determined not simply by the nominal sensitivity of the PCR platform but by the number of tumour-derived DNA molecules that remain available for interrogation after blood collection, cfDNA extraction, methylation conversion, and sample preparation. This distinction is particularly important in early-stage OC, where the tumour-derived component of total cfDNA may be limited. Serum may contain higher total cfDNA concentrations than plasma but can also contain greater amounts of non-tumour and high-molecular-weight DNA, thereby diluting low-frequency tumour-derived signals. Terp et al. (2023) further reported that recovery following bisulfite conversion can vary considerably between conversion kits, with previously reported recovery rates ranging from approximately 22% to 66% [7]. Loss of even a modest number of tumour-derived molecules may therefore materially affect detection when starting abundance is already low.

This issue applies to ddPCR as well as MSP and qMSP when the assay uses bisulfite-converted DNA. For example, the OV1 ddPCR workflow of G. Li et al. (2024) first subjected plasma-derived cfDNA to bisulfite conversion before applying methylation- and unmethylation-specific probes [16]. Thus, ddPCR can improve quantitative precision and measurement of rare targets but does not inherently eliminate losses or artefacts introduced during upstream conversion and sample preparation. For early-stage OC applications, analytical validation should therefore report not only PCR performance under ideal conditions but also cfDNA input, conversion efficiency, background methylation, limit of blank, limit of detection, and reproducibility at methylation fractions relevant to early disease. Such parameters are necessary to determine whether a targeted assay can reliably distinguish a true low-level tumour signal from technical variation or non-tumour background.

### Bisulfite sequencing (RRBS/WGBS) and ultrafast bisulfite sequencing

Bisulfite sequencing remains a widely established approach for single-base-resolution DNA methylation mapping. The method relies on conversion of unmethylated cytosines to uracil while methylated cytosines remain protected, allowing methylation status to be inferred from sequencing reads. However, conventional bisulfite treatment requires prolonged exposure to harsh chemical and thermal conditions that can cause DNA fragmentation and loss, reduce library complexity, distort GC representation, and generate uneven genomic coverage [13, 27]. Vaisvila et al. showed that these effects arise partly from bisulfite-induced DNA degradation and preferential damage of unmethylated cytosines, which can result in reduced mapping efficiency and biased sequence representation. These limitations become particularly important in liquid-biopsy applications, where the quantity of tumour-derived DNA available before conversion may already be limited.

Two major bisulfite-sequencing strategies differ primarily in genomic breadth and sequencing efficiency. Whole-genome bisulfite sequencing (WGBS) interrogates methylation across most of the genome at single-base resolution and therefore offers the broadest opportunity for unbiased discovery of differentially methylated regions. Its comprehensive coverage, however, comes at the cost of substantial sequencing depth, computational requirements, and sensitivity to bisulfite-associated DNA loss and coverage bias [13, 26]. Reduced-representation bisulfite sequencing (RRBS) instead uses restriction digestion and size selection to enrich CpG-dense genomic regions, thereby concentrating sequencing reads within a smaller fraction of the methylome and reducing sequencing requirements. This makes RRBS attractive for methylation-marker discovery from limited material, but its targeted genomic representation means that methylation alterations outside the captured CpG-rich regions may be missed [13, 26]. Thus, WGBS maximises discovery breadth whereas RRBS trades comprehensive genomic coverage for greater sequencing efficiency.

The application of RRBS to OC illustrates how genome-scale discovery can be linked to a more targeted translational assay. Marinelli et al. (2022) performed RRBS on frozen OC tissue, benign fallopian-tube epithelium, and buffy-coat DNA from cancer-free women to identify candidate OC-associated methylated DNA markers. Candidate selection incorporated methylation fold change, receiver-operating-characteristic discrimination, and low background methylation in control samples. Thirty-three candidate markers were initially identified, followed by independent tissue validation and subsequent testing of 11 selected markers in plasma [2]. This staged strategy (i.e., broad methylation discovery followed by targeted validation) demonstrates an important translational role for RRBS: genome-scale methods may be most useful for identifying candidate regions, whereas a smaller subset of markers can subsequently be transferred to analytically simpler assays for clinical testing.

A recent refinement of conventional bisulfite chemistry is ultrafast bisulfite sequencing (UBS-seq). Dai et al. (2024) developed UBS-seq using highly concentrated bisulfite reagents and elevated reaction temperatures to accelerate cytosine conversion by approximately 13-fold [28]. In contrast to the prolonged treatment required by conventional bisulfite protocols, UBS-seq substantially shortened the conversion reaction while reducing DNA degradation and background signal. The study also directly addressed a second limitation of conventional bisulfite sequencing—incomplete C-to-U conversion, which can cause unmethylated cytosines to be misclassified as methylated. Under the optimised UBS conditions, the average unconverted cytosine rate in unmethylated λ-DNA was approximately 0.06%, more than 13-fold lower than with the conventional bisulfite condition. The shortened reaction also produced less DNA degradation, which is potentially advantageous when starting DNA amounts are low.

UBS-seq is particularly relevant to liquid-biopsy research because Dai et al. demonstrated library construction from small DNA inputs, including cfDNA, and reported higher genomic coverage and less methylation overestimation than conventional BS-seq. However, UBS-seq should presently be regarded as a general analytical advance rather than an OC-specific validated assay. The study did not establish diagnostic performance in patients with OC. Its relevance to early-stage OC is therefore methodological: reducing conversion-associated DNA loss and false methylation calls may improve recovery of weak tumour-associated signals when the tumour-derived fraction of cfDNA is small, but this theoretical advantage requires direct evaluation in OC cohorts.

Overall, the choice between WGBS, RRBS, and newer bisulfite-based approaches reflects a balance between methylome coverage, input requirements, sequencing burden, and preservation of low-abundance tumour-derived DNA. Genome-wide methods are valuable for biomarker discovery, but their translational utility ultimately depends on whether identified regions can be measured reproducibly in circulating DNA at clinically relevant concentrations. Improvements in bisulfite chemistry such as UBS-seq may mitigate DNA degradation and incomplete conversion, but they do not remove the need for rigorous analytical validation of conversion efficiency, library complexity, background methylation, and limit of detection in early-stage OC samples.

### Alternative approaches: enzyme-based and enrichment-based methods

Bisulfite-free approaches have been developed to address some of the limitations associated with conventional bisulfite treatment, particularly DNA degradation and loss of library complexity at low input. These technologies are not specific to OC, but they are potentially relevant to liquid-biopsy applications in which the available tumour-derived cfDNA may be limited. Two broad strategies are of particular interest: enzymatic conversion methods, which preserve single-base methylation information without bisulfite treatment, and enrichment-based methods, which selectively capture methylated DNA fragments without nucleotide conversion.

Enzymatic methyl sequencing (EM-seq) is a bisulfite-free method designed to reduce DNA damage while retaining single-base resolution. In EM-seq, TET2 and T4-BGT first protect 5-methylcytosine (5mC) and 5-hydroxymethylcytosine (5hmC) from deamination, after which APOBEC3A converts unmodified cytosines to uracil. Vaisvila et al. (2021) demonstrated that EM-seq libraries generated from genomic DNA, cfDNA, and formalin-fixed paraffin-embedded DNA showed more even GC representation, fewer duplicates, improved CpG coverage, and greater library complexity than conventional bisulfite libraries [27]. The method remained effective at inputs as low as approximately 100 pg DNA. These characteristics are potentially advantageous for early-stage OC, where minimising loss of low-abundance tumour-derived DNA may be critical.

However, enzymatic methods should not be considered free of conversion-related error. Although EM-seq avoids incomplete bisulfite conversion, it depends on efficient enzymatic oxidation, protection, and deamination. Vaisvila et al. (2021) showed that the workflow relies on sequential TET2/T4-BGT protection and APOBEC3A-mediated deamination to distinguish modified from unmodified cytosines. Accordingly, incomplete enzymatic reactions can introduce a different class of measurement error if modified cytosines are inadequately protected or unmodified cytosines are incompletely deaminated. Dai et al. (2024) similarly noted that bisulfite-free enzymatic workflows can introduce additional operational complexity, potential reductions in conversion efficiency, and batch-to-batch variability [28]. Appropriate spike-in controls and monitoring of enzymatic conversion efficiency therefore remain necessary.

A second strategy is methylation enrichment, in which methylated DNA fragments are isolated according to their methylation density rather than converted chemically or enzymatically. This avoids conversion-associated DNA degradation but sacrifices direct single-CpG resolution because the output reflects the relative abundance of enriched methylated fragments across genomic regions. Methylated DNA can be captured using antibodies against 5mC, as in methylated DNA immunoprecipitation sequencing (MeDIP-seq), or methyl-CpG binding domain proteins, as in MBD-seq. Huang et al. (2022) developed an ultra-low-input MBD-based protocol, cfMBD-seq, by reducing the amount of MethylCap protein and incorporating methylated filler DNA to maintain an appropriate protein-to-DNA ratio during enrichment [29]. The resulting protocol enabled methylome profiling from as little as 1 ng of DNA, with genome-wide profiles remaining highly correlated with those obtained using standard MBD-seq at substantially higher input.

The use of methylated filler DNA was central to the low-input performance of cfMBD-seq. Huang et al. showed that filler DNA helped stabilise MethylCap binding and reduce non-specific capture when the amount of cfDNA was very small. At the optimised condition, the methylated spike-in control showed ≥ 99% capture specificity, while recovery of methylated control DNA was approximately 50–90% and recovery of unmethylated control DNA remained below 1%. This highlights an important feature of enrichment-based technologies: analytical performance depends not only on total DNA input but also on capture-protein concentration, methylation density, filler-DNA characteristics, and library-quality controls.

Compared with cfMeDIP-seq, cfMBD-seq produced a higher proportion of sequencing reads passing quality filters (83.15% versus 74.90%) and a lower duplicate rate (3.45% versus 12.00%) in the datasets analysed by Huang et al. (2022) It also recovered a larger proportion of reads from CpG islands (60.13% versus 38.16%). However, these differences reflect distinct enrichment preferences rather than evidence that one platform is universally superior [29]. cfMBD-seq preferentially enriches CpG-dense regions such as CpG islands, whereas cfMeDIP-seq captures relatively more signal from CpG shores and lower-density regions. Huang et al. (2022) therefore suggested that the preferred method may depend on the genomic context of interest.

This distinction is especially relevant for classifier development. Enrichment-based assays do not quantify methylation at individual CpG sites; instead, they generate regional methylation-abundance profiles that can be used to identify differentially methylated regions and provide features for downstream statistical or machine-learning models. Huang et al. explicitly noted that cfMBD-seq shares downstream analytical workflows with cfMeDIP-seq for DMR discovery and machine-learning analyses. Such approaches may therefore be suitable for exploratory methylome-wide classifier development when biological interpretation of single CpG sites is not the primary objective.

Nevertheless, the relevance of cfMBD-seq to OC remains methodological rather than clinically validated. The Huang et al. study was principally a technology-development and optimisation study and did not evaluate diagnostic performance in patients with OC. The authors themselves acknowledged that further validation in patient samples is required to determine whether cfMBD-seq can distinguish tumour-derived methylation signals from the high background of non-tumour cfDNA. Similarly, EM-seq has demonstrated favourable analytical characteristics across low-input samples but has not yet established OC-specific screening performance. These platforms should therefore be presented as promising technologies for biomarker discovery and assay development rather than as validated approaches for early OC detection.

Overall, enzyme-based and enrichment-based approaches address different limitations of conventional bisulfite sequencing. EM-seq preserves single-base resolution while reducing bisulfite-associated DNA damage, whereas cfMBD-seq and related enrichment methods avoid conversion entirely and can tolerate very low DNA input at the cost of base-level resolution. Their potential value in early-stage OC will depend on whether these analytical advantages translate into reproducible detection of tumour-derived methylation signals at very low abundance, across independent cohorts and clinically relevant pre-analytical conditions.

### Quality control and pre-analytical considerations

Pre-analytical handling and analytical quality control are particularly important for methylation-based liquid biopsy because the measured signal depends not only on the biological abundance of tumour-derived DNA but also on how much of that signal is preserved through blood collection, plasma or serum preparation, cfDNA extraction, methylation processing, and downstream analysis. This is especially relevant to early-stage OC, where loss or dilution of a small number of tumour-derived DNA molecules may substantially affect apparent biomarker sensitivity. Considerable heterogeneity in these procedures has been reported across published OC methylation studies, which may contribute to between-study differences in diagnostic performance [7].

Blood collection and sample processing represent the first major source of variation. Delayed processing can promote leukocyte lysis and release of high-molecular-weight genomic DNA, increasing the non-tumour DNA background and thereby reducing the relative abundance of tumour-derived cfDNA. Terp et al. (2023) found that only six of 29 reviewed OC methylation studies reported the interval between blood collection and processing, while blood collection tubes, centrifugation protocols, sample volumes, and storage conditions also varied considerably [7]. Double centrifugation is generally used to reduce residual cellular material, yet only four studies in that review reported using a double-centrifugation protocol. Plasma or serum input volumes ranged from approximately 0.1 to 10 mL, introducing an additional source of variability in the number of cfDNA molecules available for analysis. Consequently, studies should report the blood collection tube, interval to processing, centrifugation conditions, storage temperature and duration, and starting plasma or serum volume rather than describing the specimen simply as “blood-derived cfDNA”.

The choice of plasma versus serum and cfDNA extraction method also requires consideration. Although serum may contain a higher total concentration of cfDNA, tumour-derived DNA can be diluted by greater amounts of non-tumour DNA released during clotting and cellular disruption, potentially reducing detection of low-frequency tumour signals. Extraction efficiency is similarly method dependent. Terp et al. reported that only 11 of 29 studies used extraction kits specifically developed for cfDNA; use of protocols designed for high-molecular-weight genomic DNA may reduce recovery of short cfDNA fragments and thereby contribute to reduced analytical sensitivity. For early-stage applications, reporting total cfDNA yield alone is therefore insufficient; studies should also document extraction method and assess contamination by high-molecular-weight genomic DNA where possible.

Quality control must then be adapted to the methylation chemistry used. For bisulfite-based methods, incomplete conversion of unmethylated cytosines can generate false methylation calls, whereas excessively harsh conversion conditions can cause DNA degradation and loss. Dai et al. (2024), for example, used unmethylated λ-DNA and structured control DNA to quantify residual unconverted cytosine and showed that UBS-seq reduced the average unconverted cytosine background to approximately 0.06%, substantially below that observed with conventional bisulfite treatment. Conversion controls should therefore accompany low-input bisulfite assays so that apparent low-level methylation can be distinguished from incomplete conversion. Enzymatic methods require analogous but chemistry-specific controls rather than conventional “bisulfite conversion” controls. In EM-seq, Vaisvila et al. incorporated both unmethylated λ-DNA and CpG-methylated pUC19 DNA during library construction to monitor the behaviour of unmethylated and methylated cytosines. Such controls are necessary because inadequate enzymatic protection or deamination can also introduce erroneous methylation calls even though bisulfite is not used.

Library quality and sequencing performance should also be reported explicitly for sequencing-based assays. Relevant parameters include starting DNA input, library yield, duplication rate, proportion of usable or mapped reads, coverage depth, GC representation, and reproducibility between technical replicates. These measures are especially important when comparing low-input technologies because high nominal sequencing depth may provide limited additional information if much of the library consists of duplicates or biased genomic representation. Vaisvila et al. demonstrated that low-input EM-seq libraries maintained relatively even GC representation, although the lowest-input libraries required additional PCR cycles and showed reductions in unique reads. Similarly, Dai et al. showed that uneven or incomplete conversion can generate regionally concentrated false-positive methylation signals, highlighting the need to examine coverage and background across the genome rather than relying solely on average conversion rates.

Enrichment-based approaches require an additional set of capture-specific quality controls. In cfMBD-seq, Huang et al. (2022) used methylated and unmethylated spike-in controls to assess enrichment efficiency and recommended qPCR-based assessment before sequencing; under optimised conditions, capture specificity for methylated control DNA was ≥ 99%, recovery of methylated control DNA was approximately 50–90%, and recovery of unmethylated control DNA remained below 1% [29]. Sequencing-level metrics were also informative: when cfMBD-seq was compared with cfMeDIP-seq, differences were observed in the proportion of reads passing filters, duplicate rates, and genomic regions preferentially enriched. Huang et al. (2022) further estimated that approximately 30 million mapped reads were required to obtain a saturated and reproducible cfMBD-seq coverage profile under their experimental conditions. These findings emphasise that low-input compatibility alone is not sufficient; enrichment efficiency and library complexity must also remain stable across samples and batches.

Finally, assay thresholds, batch effects, and validation procedures are critical when analytical measurements are translated into diagnostic classifications. Even when the same gene is interrogated using nominally similar qMSP methods, targeting different CpG regions can produce markedly different sensitivities, as demonstrated for RASSF1A in the OC literature. Absolute methylation cut-offs derived from one cohort should therefore not be assumed to remain valid when primer sets, extraction procedures, conversion chemistry, instruments, or patient populations change. For targeted assays, thresholds should ideally be prespecified and subsequently evaluated in independent cohorts rather than repeatedly optimised to maximise discrimination within the discovery dataset. For sequencing-derived and machine-learning classifiers, the same principle extends to feature normalisation and batch correction: model performance should be evaluated after the feature set and decision threshold have been locked and, where possible, tested across independently collected samples and laboratory sites.

Taken together, standardisation of pre-analytical and analytical procedures is essential for distinguishing true biological variation from technical variability in cfDNA methylation studies. At a minimum, future OC studies should report specimen type and volume, blood collection tube, processing interval, centrifugation and storage conditions, cfDNA extraction method and yield, DNA input into the methylation assay, conversion or enrichment controls, library-complexity and sequencing metrics, assay-specific limits of detection, batch-control procedures, and the method used to establish diagnostic thresholds. Such reporting is particularly important for early-stage OC, where technical loss or background contamination may have a disproportionate effect on detection of low-abundance tumour-associated methylation signals.

## Translational barriers to DNA methylation–based early detection of ovarian cancer

Despite encouraging advances in methylation biomarker discovery and assay development, substantial barriers remain before these approaches can be translated into clinically effective early-detection strategies for OC. These challenges extend beyond diagnostic accuracy and include biological heterogeneity, variability across pre-analytical and analytical workflows, limited validation in intended-use populations, uncertainty regarding clinical utility, and the practical requirements of healthcare-system implementation. Importantly, promising performance in retrospective studies does not necessarily translate into effective screening in asymptomatic populations. Successful clinical translation will therefore require coordinated consideration of biological validity, analytical reproducibility, rigorous prospective validation, patient-centred outcomes, and feasibility of implementation at scale.

### Biological signal definition and disease heterogeneity

A fundamental translational challenge is defining a methylation signal that is sufficiently sensitive to early OC, specific to the relevant disease process, and representative of its biological heterogeneity. Early methylome-discovery studies were constrained by the genomic coverage of the available platforms. For example, initial OC datasets generated using the Illumina HumanMethylation27 array interrogated fewer than 0.1% of genomic CpG sites, whereas subsequent 450 K and MethylationEPIC arrays progressively expanded coverage; the latter interrogates approximately 850,000 CpGs, representing around 3% of the genomic CpG repertoire [30]. Genome-scale sequencing now enables substantially broader interrogation, but studies continue to differ in the regions and genomic contexts captured by their respective platforms. This matters because cancer-associated methylation is not restricted to promoter CpG islands but occurs across promoters, gene bodies, enhancers, intergenic regions, and other regulatory elements [1, 30]. Consequently, apparently discordant biomarker findings may partly reflect differences in genomic coverage and target selection rather than true biological inconsistency.

Biological heterogeneity presents an additional challenge. OC comprises several histological entities with distinct cells of origin, molecular drivers, and epigenetic landscapes. HGSC dominates many biomarker-discovery cohorts, while endometrioid, clear-cell, mucinous, and low-grade serous carcinomas are substantially less represented. Liberto et al. emphasised that these subtypes differ in clinicopathological and molecular characteristics, creating additional challenges for development of broadly applicable early-detection assays. Evidence from methylome profiling further suggests that these differences extend to DNA methylation: OC tumours have been classified into distinct methylation subgroups, and genome-wide analyses have reported greater overall hypermethylation in HGSC than in lower-grade epithelial ovarian tumours [1, 30]. A signature derived predominantly from HGSC should therefore not be assumed to detect other histotypes with equivalent sensitivity.

This limitation can be observed in recent cfDNA classifier studies. Although G. Li et al. (2024) evaluated several histological subtypes, early-stage subgroup sizes varied considerably, limiting precision of subtype-specific performance estimates. Similar heterogeneity has been observed across the wider blood-based methylation literature: Guo et al. (2021) found that serous tumours constituted the majority of cases in most studies, whereas endometrioid and mucinous cancers were represented much less frequently; moreover, a median of 77.5% of cases were stage III–IV [10]. Thus, a biomarker may appear broadly representative of “ovarian cancer” while in practice being developed primarily from advanced serous disease. Future validation should therefore report performance separately by histotype and stage rather than treating OC as a biologically homogeneous outcome.

The composition of circulating DNA introduces a second level of biological heterogeneity. Plasma cfDNA is not synonymous with circulating tumour DNA (ctDNA); rather, it contains fragments released from multiple non-malignant tissues in addition to the tumour-derived component. M.C. Liu et al. (2020) illustrated this directly in the CCGA study: cfDNA from individuals without cancer originated from cells throughout the body, including white blood cells, whereas plasma from patients with cancer contained a mixture of tumour-derived and non-tumour fragments [15]. In early-stage disease, where fewer tumour-derived molecules may be available for interrogation than in advanced disease, this background can reduce the effective signal-to-noise ratio. The translational challenge is therefore not simply to identify CpGs that are highly methylated in tumour tissue, but to identify patterns that remain detectable against the much larger background of non-tumour cfDNA.

A related issue is cancer specificity. Aberrant methylation is a common feature of malignancy, and many individual methylated loci are not unique to OC. Guo et al. (2021), for example, concluded that no single gene had been identified as being predominantly methylated only in OC, supporting the use of combinations of methylation targets rather than reliance on individual loci [10]. A blood test that detects a generic malignant methylation pattern without identifying its anatomical origin could therefore generate a positive result from a non-OC. This issue is particularly relevant when assays are developed using only healthy controls, because such designs demonstrate cancer-versus-non-cancer discrimination but provide limited evidence of ovarian-cancer specificity.

Methylation-based tissue-of-origin (TOO) classification may partly address this problem because epigenetic patterns retain information relating to cellular differentiation and tissue identity. In the multi-cancer CCGA study, targeted cfDNA methylation analysis generated a tissue-of-origin prediction for 96% of validation samples with a cancer-like signal and correctly localised the predicted origin in 93% of those cases [15]. Importantly, however, this was a multi-cancer classifier and should not be interpreted as evidence that any individual OC methylation marker is intrinsically ovary specific. TOO localisation instead depends on recognising multivariate methylation patterns across many informative genomic regions. Liu et al. themselves emphasised that accurate tissue localisation is clinically important because a positive circulating cancer signal without localisation could lead to extensive and potentially unnecessary diagnostic investigations. OC-specific assays should therefore be evaluated not only for their ability to distinguish cancer from healthy controls but also for their ability to discriminate OC from other malignancies that may generate overlapping circulating methylation signals.

Specificity must also be established against benign gynaecological conditions. Women undergoing evaluation for an adnexal or pelvic mass may have endometriomas, ovarian cysts, cystadenomas, fibroids, endometriosis, or other non-malignant conditions that can overlap clinically with OC. In the review by Guo et al. (2021), only 10 studies included a distinct group of patients with benign ovarian or pelvic masses, despite the greater clinical relevance of these comparators compared with healthy volunteers [10]. Studies incorporating benign masses provide a more stringent test of disease specificity because they address whether the methylation signal distinguishes malignant from non-malignant gynaecological pathology rather than merely distinguishing patients with established cancer from healthy individuals. Recent studies have increasingly adopted this approach, including Zhou et al. (2025), which used patients with benign pelvic masses as the comparator group during plasma methylome discovery [23]. Nevertheless, benign comparator groups remain heterogeneous and relatively small across much of the literature.

Taken together, the biological signal required for early OC detection must satisfy several criteria simultaneously: it should be detectable at low tumour-derived cfDNA abundance, remain informative across early disease stages, capture or appropriately account for histological heterogeneity, discriminate malignant from benign gynaecological conditions, and distinguish OC-associated signals from those originating from other cancers. Meeting these requirements is unlikely to depend on a single universally methylated locus. Instead, robust translation will probably require carefully selected multi-region signatures in which individual features contribute complementary information about malignancy, disease subtype, and tissue of origin, followed by validation in populations that reflect the intended clinical setting.

### Pre-analytical and analytical variability

Even when biologically informative methylation signals have been identified, their translation into reproducible diagnostic assays is complicated by substantial variability across the pre-analytical and analytical workflow. Published OC methylation studies differ in specimen type, blood collection tubes, time to processing, centrifugation protocols, sample volume, storage conditions, cfDNA extraction methods, methylation pretreatment, and downstream analytical platforms [5, 7, 26]. Terp et al. (2023) demonstrated considerable heterogeneity in these parameters across 29 blood-based OC methylation studies: only six reported the interval between blood collection and processing, only four used a double-centrifugation protocol, and starting serum or plasma volumes ranged from 0.1 to 10 mL [7]. Such variation is especially consequential for early-stage disease because differences in the number of cfDNA molecules recovered, or contamination with high-molecular-weight DNA from lysed blood cells, can materially change the effective abundance of a low-level tumour-derived methylation signal.

Differences in cfDNA extraction and methylation pretreatment further complicate comparison between studies. Only 11 of the 29 studies evaluated by Terp et al. used extraction kits specifically designed for circulating DNA; others employed genomic blood-DNA kits, viral nucleic-acid kits, phenol/chloroform extraction, or other protocols. Methods intended for high-molecular-weight genomic DNA may recover fragmented cfDNA less efficiently, potentially reducing the number of tumour-derived molecules entering the methylation assay. Serum and plasma are also not analytically interchangeable: although serum may contain greater total cfDNA concentrations, tumour-derived cfDNA can be diluted by additional non-tumour DNA released during clotting and cellular disruption [7]. These factors mean that an apparently identical methylation marker may be evaluated against substantially different biological inputs across studies.

Methylation-processing chemistry introduces a further layer of variability. Bisulfite conversion can cause DNA degradation and loss, and recovery differs according to the conversion protocol; Terp et al. cites reported recovery rates ranging from approximately 22% to 66% across bisulfite-conversion kits. Incomplete bisulfite conversion may additionally cause unmethylated cytosines to be misclassified as methylated. Enzymatic and enrichment-based methods avoid some of these limitations but should not be considered analytically error-free. Enzymatic approaches depend on efficient protection, oxidation, and deamination reactions, whereas enrichment-based assays introduce biases related to methylation density, genomic context, and capture efficiency. Thus, different platforms may interrogate biologically different fractions of the methylome even when they are nominally being used to address the same diagnostic question. These method-specific effects complicate direct comparison of sensitivity or specificity between studies performed using different assay chemistries.

Variability can persist even within the same broad analytical platform. Targeted methods such as MSP, qMSP, and ddPCR interrogate predefined CpG sites, meaning that primer and probe location, reference regions, methylation-normalisation procedures, and positivity thresholds can materially influence the resulting diagnostic classification. Terp et al. noted that substantial differences in reported performance could occur even among studies examining the same gene using broadly similar methylation-PCR approaches, partly because different CpG regions were targeted. Consequently, a methylation threshold optimised within one discovery cohort should not automatically be transferred to another population or laboratory. Re-optimising the threshold in every new cohort, however, creates a different problem because it can inflate apparent diagnostic performance and obscure whether the assay itself is genuinely reproducible.

The translational requirement is therefore not simply to identify the analytically “best-performing” platform, but to establish an end-to-end standardised workflow that produces consistent results across collection sites, laboratories, patient populations, and time. At minimum, assay development should prespecify sample type and volume, blood-processing conditions, cfDNA extraction and methylation-treatment protocols, analytical quality-control criteria, target regions, normalisation procedures, and decision thresholds. Once established, these parameters should be locked before independent validation, with inter-run, inter-operator, and ideally inter-laboratory reproducibility evaluated before clinical performance is interpreted. The absence of a standardised procedure for liquid-biopsy methylation analysis remains an important barrier to clinical implementation [26].

### Limitations in clinical validation

A major bottleneck in translating DNA methylation biomarkers into early OC detection is the limited depth and clinical relevance of existing validation. Much of the published evidence has been generated from retrospective or case–control cohorts in which blood was collected from patients with already diagnosed cancer and compared with healthy individuals or selected benign controls. Cohort sizes have also generally been modest. In the systematic review by Terp et al. (2023), training cohorts contained only 16–91 °C cases, validation cohorts contained 8–43 cases, and fewer than half of the included studies enrolled at least 50 cancer cases. Advanced-stage disease was represented in every study reporting stage information, whereas the number of genuinely early-stage cases was often small [7]. Consequently, overall diagnostic performance may be disproportionately influenced by patients with greater tumour burden and more abundant circulating tumour-derived DNA rather than reflecting the intended challenge of detecting FIGO stage I–II disease.

Case–control designs are particularly vulnerable to spectrum bias when clinically obvious cancers are compared with healthy controls who differ substantially from the population in which the test would eventually be used. Under these circumstances, sensitivity, specificity, and AUC may overstate discrimination achievable in an asymptomatic or clinically heterogeneous screening population. Disease prevalence presents a related but distinct issue: although sensitivity and specificity are not mathematically determined by prevalence, positive and negative predictive values are. Consequently, PPV estimates derived from case–control cohorts enriched with OC cannot be extrapolated to average-risk population screening. This distinction is particularly important for OC because its low prevalence imposes stringent specificity requirements and makes even a small false-positive rate clinically consequential.

A further limitation is the small number of early-stage cases within studies reporting apparently high stage-specific performance. For example, Marinelli et al. (2022) detected all five stage I–II HGSC cases included in their plasma cohort, demonstrating proof-of-concept but not providing a sufficiently precise estimate of early-stage sensitivity because the denominator was only five patients [2]. Similar caution applies to studies in which stage-stratified performance is based on only a handful of cases. Reporting the number of stage I–II cases alongside the corresponding sensitivity is therefore essential; percentages presented without their denominators can give an exaggerated impression of evidential robustness.

The difference between diagnostic validation and true early-detection validation is illustrated particularly clearly by Herzog et al. (2024). In a diagnostic cohort of 27 °C cases and 41 controls that included women with benign pelvic pathology, the three-region WID-cfOC methylation score achieved 97.6% specificity and sensitivities of 66.7% for all cancers and 80% for high-risk cancers [24]. When the same score was subsequently evaluated using samples collected before diagnosis from the UK Familial Ovarian Cancer Screening Study, however, sensitivity was substantially lower. Among high-risk cancers with relatively lower genomic-DNA contamination, sensitivity was 27.3%, increasing to 33.3% for samples collected within one year of diagnosis, despite specificity remaining 100%. Neither methylation nor CA125 detected any stage I cancer in the diagnostic cohort, and only one stage II cancer was identified; most stage III–IV cancers were detected. Although archival sample quality and genomic-DNA contamination contributed to the lower pre-diagnostic performance, the findings demonstrate why successful discrimination of established cancer from controls should not be equated with effective preclinical detection.

Validation should therefore be considered as a hierarchy rather than a binary characteristic. Biomarker discovery establishes an association with disease; internal cross-validation or random train–test splitting evaluates performance within the source population; external validation examines transportability to an independently collected cohort; pre-diagnostic validation tests whether the signal is present before clinical diagnosis; and prospective intended-use studies determine performance under the conditions in which screening would actually occur. These stages are not interchangeable. Herzog et al. noted, for example, that although Marinelli et al. and Liang et al. (2023) performed independent validation, neither had validated its method in samples predating diagnosis [31]. Thus, describing an assay simply as “validated” can conceal important differences in the strength of supporting evidence.

This distinction is equally important for machine-learning classifiers. Internal partitioning of a retrospective dataset can provide useful evidence against gross overfitting but does not establish generalisability across institutions, populations, or assay conditions. Conversely, independent external validation can reveal deterioration after model simplification. Gonzalez Bosquet et al. (2025), for example, achieved near-perfect performance during development of methylation-based HGSC models, but external validation of the simplified nine-probe classifier yielded an AUC of 0.84 (95% CI 0.76–0.93), compared with 0.98 for the much larger 11,167-probe model. The investigators themselves emphasised the need for subsequent validation in blood, across diverse populations and disease stages, and against controls including women with benign pelvic masses. Such findings highlight the potential trade-off between model parsimony, transportability, and diagnostic accuracy and reinforce the need to assess feature stability outside the development population.

Clinical validation should therefore move beyond reporting a single AUC or an overall sensitivity/specificity pair. Studies intended to support early detection should prespecify assay thresholds and primary endpoints; report cohort size, FIGO stage and histotype distribution; provide stage I–II performance with denominators and confidence intervals; include clinically relevant benign comparator groups; and distinguish clearly between internal, external, pre-diagnostic, and prospective validation. Ultimately, the strongest evidence will require prospective evaluation in asymptomatic or clearly defined high-risk populations using samples collected and processed according to the intended clinical workflow. Until such evidence becomes available, the high diagnostic performance reported by many methylation studies should be regarded as evidence of biomarker potential rather than established screening effectiveness.

### Clinical utility and patient-centred considerations

fit. In UKCTOCS, longitudinal CA125 assessment followed by transvaginal ultrasound produced a reduction in advanced-stage diagnoses, but this stage shift did not translate into a demonstrated survival benefit; consequently, this strategy is not recommended for population screening. Similarly, previous CA125- and ultrasound-based screening has illustrated the consequences of false-positive findings and the difficulty of maintaining adequate specificity while detecting early disease. Bast et al. reported PPVs of only 3.7% for elevated CA125 and 1% for abnormal transvaginal ultrasound in PLCO; requiring both tests to be abnormal increased PPV to 23.5% but would have missed approximately 80% of cancers. These experiences establish an important benchmark for methylation-based assays: clinical utility should ultimately be assessed through meaningful outcomes such as reduction in late-stage disease and, ideally, OC-specific mortality, rather than diagnostic accuracy alone.

The potential role of methylation assays may therefore lie not only in replacing established biomarkers but also in complementing them within a multistep screening strategy. However, evidence of additive benefit with CA125 should be interpreted according to both sensitivity and specificity. In G. Li et al. (2024), combining MethylBERT-EOC with CA125 increased overall sensitivity from 92.47% to 95.68%, and among 183 early-stage cases increased sensitivity from 82.51% to 89.62% [16]. This gain was accompanied by a reduction in specificity from 97.36% to 93.55%. At a more stringent specificity of 99.2%, the MethylBERT model alone detected only 63.01% of early-stage cases, illustrating how apparently strong overall performance can diminish when thresholds are adjusted toward those required for screening. Therefore, increases in sensitivity obtained by adding CA125 should not automatically be described as improved screening performance if the resulting loss of specificity would produce an unacceptable number of false-positive investigations.

A similar sensitivity–specificity trade-off was observed by Herzog et al. (2024). In their diagnostic cohort, the WID-cfOC methylation score achieved 77.8% sensitivity and 97.4% specificity for high-risk cancers among patients with available CA125 results, compared with 83.3% sensitivity and 87.2% specificity for CA125 [24]. Combining the tests increased sensitivity to 94.4% but reduced specificity to 87.2%, and the differences were not statistically significant because of the small sample size. More importantly, in the pre-diagnostic UKFOCSS subset with lower genomic-DNA contamination, cfDNA methylation achieved 22.2% sensitivity and CA125 44.4%, while their combination remained at 44.4% sensitivity, each at 100% specificity within this small subset. These findings reinforce that additive benefit demonstrated in patients with established disease may not persist in samples collected before clinical diagnosis.

The downstream pathway following a positive methylation result is equally important from a patient perspective. A blood-based test is minimally invasive, but its harms are determined partly by what follows a positive result. Because very small early OC lesions may not be visible on conventional imaging, a positive molecular signal may trigger repeat blood tests, transvaginal ultrasound, cross-sectional imaging, specialist consultation, or potentially surgical investigation. The UKCTOCS experience illustrates the difficulty of resolving discordant molecular and imaging findings: among women with an abnormal ROCA result but CA125 below 35 U/mL at the relevant annual screen, the median interval between initial positivity and surgery was approximately 30 weeks, partly reflecting repeated testing and clinicians’ reluctance to operate without a visible tumour. A future methylation-based screening strategy therefore requires a clearly defined diagnostic algorithm for managing positive, negative, and indeterminate results rather than viewing the blood test as an isolated intervention.

Clinical utility should ultimately be evaluated at the level of the complete screening pathway. Prospective studies should determine not only sensitivity and specificity but also PPV, number needed to investigate, number of imaging procedures and operations generated per cancer detected, interval from molecular positivity to diagnosis, stage distribution at diagnosis, and downstream clinical outcomes. Longitudinal follow-up will also be necessary to determine whether low-level methylation signals that precede diagnosis represent progressive malignancy requiring intervention or transient/background signals that do not warrant immediate action. Until such evidence is available, methylation-based assays should be regarded as promising components of future OC detection pathways rather than clinically validated screening tests.

### Implementation and health system challenges

Implementation of DNA methylation–based early detection within healthcare systems will require assays that are scalable, reproducible, timely, and affordable for repeated use in the intended population. Genome-wide methylation profiling and high-dimensional classifiers are valuable for biomarker discovery but may be difficult to deploy routinely because of sequencing requirements, specialised bioinformatics, and assay complexity. Translation may therefore require conversion of complex signatures into smaller targeted assays that retain acceptable diagnostic performance while reducing analytical burden. G. Li et al. (2024), for example, translated the sequencing-derived OV1 methylation marker into a targeted ddPCR assay as a simpler and potentially more practical testing format [16].

Implementation must also consider the resources generated by positive test results. In the prospective high-risk cohort reported by G. Li et al. (2024), OV1 ddPCR combined with CA125 was used as a first-line test, with positive participants proceeding to transvaginal ultrasonography, followed by magnetic resonance imaging and possible surgical assessment when findings remained suspicious. Of 2,117 participants, 314 screened positive at the first stage, while only four cancers among these participants were confirmed during the study period. Although interpretation is limited by short follow-up and incomplete confirmation of disease status, these findings illustrate how insufficient specificity can generate substantial downstream imaging and clinical workload [16].

The optimal position of methylation testing within the OC detection pathway also remains uncertain. Potential roles include first-line screening, adjunctive testing with CA125, risk refinement before imaging, or second-line assessment following an abnormal conventional test. Each role imposes different requirements for sensitivity, specificity, turnaround time, and cost. Economic evaluation should therefore consider not only the price of the molecular assay but also repeat testing, imaging, specialist consultations, surveillance, and surgery generated by positive results. Existing OC screening analyses demonstrate that cost-effectiveness is highly dependent on test performance, disease prevalence, and downstream resource utilisation [30].

Finally, routine implementation will require standardised sample handling, assay procedures, quality-control criteria, and diagnostic thresholds across laboratories. For classifier-based tests, computational preprocessing, feature sets, algorithm versions, and decision thresholds must also remain reproducible. Accordingly, future prospective studies should evaluate methylation assays as components of complete clinical pathways, incorporating diagnostic accuracy, resource utilisation, cost-effectiveness, accessibility, and feasibility of delivery at scale.

## Future directions and research priorities

Advancing DNA methylation–based approaches toward clinically meaningful early detection of OC will require coordinated progress in biomarker selection, assay development, clinical validation, and data integration. Future research should move beyond maximising diagnostic performance within individual discovery cohorts and instead prioritise reproducibility, early-stage sensitivity at clinically relevant specificity, and feasibility within intended-use populations. Particular emphasis should be placed on defining minimal and robust methylation signatures, optimising low-input cfDNA technologies, conducting prospective and longitudinal validation, integrating complementary biomarkers, and developing transparent data-driven models that can be translated into scalable clinical workflows.

### Defining minimal and robust methylation signatures

A key research priority is to define minimal methylation signatures that retain clinically relevant early-stage discrimination while reducing assay complexity. In practical terms, a minimal signature should comprise the smallest fixed set of CpG sites or differentially methylated regions (DMRs) that can be transferred to a targeted assay and maintain reproducible performance, particularly for FIGO stage I–II disease, at a prespecified high specificity. Importantly, increasing the number of methylation features does not necessarily improve early detection. N. Li et al. (2022), for example, identified 1,272 DMRs and developed a blood-based machine-learning classifier with an AUC of 0.94 in training, yet the independent test cohort detected only 44.4% of stage I–II cancers (*n* = 9) [32]. This illustrates the need to optimise signatures for early-stage performance rather than overall classification accuracy alone. The study nevertheless demonstrates the feasibility of distinguishing malignant from benign or healthy ovarian conditions using blood-based methylation profiling.

Marker selection should therefore prioritise features that remain stable across independent cohorts, histological subtypes, populations, and analytical platforms, rather than those selected primarily because they maximise discrimination within a discovery dataset. Recurrent methylation changes may indicate greater biomarker robustness, although recurrence alone should not be interpreted as evidence of a causal role in tumorigenesis. Where possible, integration with gene-expression or functional data may strengthen biological plausibility and improve interpretability.

Genome-wide discovery will remain important for identifying candidates from which smaller signatures can subsequently be derived. Zhou et al. (2025), for example, identified 35 differentially methylated genes and subsequently narrowed these to candidate sites within NBL1 and CASZ1; NBL1 hypermethylation showed concordance between blood and tissue and was inversely associated with gene expression [23]. However, the study included only 10 patients with EOC and 10 with benign pelvic masses, and therefore remains discovery-stage rather than evidence for a clinically established minimal signature. Future studies should consequently emphasise feature reduction followed by independent validation, rather than progressively increasing signature complexity.

### Optimisation of analytical platforms for low-input cfDNA

Future assay development should prioritise reliable measurement of methylation when the tumour-derived component represents only a small fraction of total cfDNA. Genome-scale approaches such as WGBS and RRBS remain valuable for biomarker discovery, but sequencing burden and bisulfite-associated DNA degradation can limit their practicality for repeated clinical testing. Newer low-input adaptations may mitigate some of these limitations; for example, modified RRBS approaches have been developed to increase regulatory-region coverage while accommodating smaller DNA inputs [13]. Once robust markers are identified, translation to targeted platforms such as qMSP or ddPCR may provide a more scalable strategy. G. Li et al. (2024), for example, transferred the sequencing-derived OV1 marker to a targeted ddPCR assay, illustrating this discovery-to-targeted-assay approach [16].

Further evaluation of technologies that minimise conversion-associated DNA loss is also warranted. EM-seq has demonstrated reliable methylation profiling at DNA inputs down to approximately 100 pg, with favourable library coverage and GC representation compared with conventional WGBS [27]. Improved bisulfite approaches such as UBS-seq and low-input enrichment methods may provide additional options, although their advantages require validation specifically in OC cohorts.

Importantly, optimisation should focus not only on nominal DNA-input requirements but on effective analytical sensitivity across the complete workflow. Future studies should establish limits of blank and detection, recovery of low-abundance methylated molecules, reproducibility across laboratories, and performance using cfDNA concentrations and tumour fractions representative of early-stage OC.

### Prospective and longitudinal clinical validation

Prospective and longitudinal validation represents one of the most important priorities for translating methylation biomarkers into early OC detection. Most available evidence remains derived from retrospective case–control cohorts, which may not reflect performance in the intended screening population. Future studies should therefore evaluate locked methylation signatures and prespecified thresholds in asymptomatic or clearly defined high-risk populations, with adequate representation of FIGO stage I–II disease and clinically relevant benign conditions.

Prediagnostic samples are particularly valuable because they establish whether a methylation signal is detectable before clinical diagnosis, rather than only after an established tumour is present. Herzog et al. (2024), for example, evaluated cfDNA methylation in UKFOCSS samples collected before diagnosis and detected 33.3% of high-risk cancers in samples obtained within one year of diagnosis, although the study was limited by small numbers and archival sample quality. The authors also noted that previous independently validated OC methylation studies had generally not tested samples predating diagnosis [24].

Longitudinal studies should additionally incorporate repeated sampling and sufficiently long follow-up to determine whether low-level methylation signals persist, increase before diagnosis, or represent transient background variation. G. Li et al. (2024) provided an important step by evaluating OV1 and CA125 prospectively in 2,117 high-risk women, but the authors acknowledged that short follow-up and reliance on imaging to classify many participants could affect estimates of sensitivity and specificity [16]. Future studies should therefore include longer follow-up, predefined clinical endpoints, histological confirmation where available, and evaluation of stage-specific sensitivity, specificity, PPV, and downstream clinical outcomes.

### Integration with established biomarkers and multi-omics data

Future early-detection strategies are likely to benefit from combining methylation signals with complementary biomarkers rather than relying on a single molecular modality. CA125 is the most obvious partner because it is already embedded in OC diagnostic pathways, but its additive value should be assessed according to both sensitivity and specificity. In G. Li et al. (2024), combining MethylBERT-EOC with CA125 increased early-stage sensitivity from 82.51% to 89.62%, but reduced overall specificity from 97.36% to 93.55% [16]. Herzog et al. (2024) similarly reported that combining cfDNA methylation with CA125 increased sensitivity for high-risk cancers to 94.4%, but specificity fell to 87.2% [24]. Future combinatorial models should therefore demonstrate that any improvement in sensitivity remains clinically acceptable at the very high specificity required for screening.

Beyond CA125, integration of methylation with other molecular signals may help address tumour heterogeneity and tissue-of-origin uncertainty. Potential complementary features include somatic mutations, copy-number alterations, transcriptomic or microRNA profiles, and protein biomarkers. Multi-analyte approaches have already demonstrated the feasibility of combining genomic and protein information for cancer detection, although their performance and clinical value remain dependent on cancer stage and intended-use population [25, 30].

Future multi-omics models should therefore prioritise complementary biological information rather than simply increasing the number of features. Added molecular layers should be retained only when they provide reproducible incremental value beyond methylation and established biomarkers, while preserving interpretability, assay feasibility, and clinically appropriate specificity.

### Application of artificial intelligence and data-driven frameworks

Artificial intelligence and machine-learning approaches are well suited to high-dimensional methylation data because they can model interactions among multiple CpG features that may not be captured by simpler statistical methods. G. Li et al. (2024), for example, pretrained MethylBERT using methylation data from more than 110,000 cancer samples and subsequently applied the model to 493 °C-associated cfDNA methylation markers [16]. In the held-out validation dataset, MethylBERT achieved 89.24% sensitivity and 94.39% specificity overall, with 79.45% sensitivity among 73 early-stage cases; using the same dataset, a conventional LASSO model achieved 67.12% early-stage sensitivity. These findings support further investigation of data-driven models for extracting informative patterns from complex methylation datasets.

However, increasing computational complexity does not necessarily improve clinical generalisability. Future AI models should prioritise feature stability, transparent preprocessing and model development, and validation across independent populations and assay platforms. Gonzalez Bosquet et al. (2025) illustrates this challenge: a nine-probe model achieved perfect discrimination during development, but its AUC declined to 0.84 (95% CI 0.76–0.93) when evaluated in an independent external dataset. The authors also acknowledged the “black-box” nature of deep-learning feature selection and the need for validation in blood-based samples, diverse populations, different disease stages, and benign pelvic controls [17].

Future AI frameworks should therefore aim not simply to maximise AUC but to identify reproducible and clinically transferable decision models, ideally using locked feature sets and thresholds followed by external and prospective intended-use validation. Where possible, model simplification and biological interpretability should be balanced against performance so that computational advances ultimately support, rather than hinder, assay standardisation and clinical implementation.

## Conclusion

DNA methylation represents a promising biomarker class for early OC detection because tumour-associated epigenetic alterations can be detected in circulating DNA and may provide information beyond conventional serum biomarkers. The field has progressed from individual candidate genes toward multi-locus signatures, genome-wide discovery, and machine-learning classifiers, with several studies demonstrating encouraging discrimination of early-stage disease. However, no methylation assay has yet demonstrated the combination of robust stage I–II sensitivity, very high specificity, histotype coverage, analytical reproducibility, and prospective intended-use validation required for population screening. Much of the current evidence remains derived from retrospective case–control studies, often with small early-stage subsets, predominantly serous disease, and healthy rather than clinically relevant comparator populations. Performance must therefore be interpreted in relation to study design, disease stage, assay methodology, comparator composition, and level of validation rather than by overall AUC or sensitivity alone.

Future progress will depend less on increasing biomarker or computational complexity and more on identifying minimal, reproducible methylation signatures that can be measured reliably in low-input cfDNA and retain performance across populations, histological subtypes, and laboratories. Standardised pre-analytical and analytical workflows, locked assay thresholds and classifier features, and prospective longitudinal evaluation in asymptomatic or clearly defined high-risk populations will be essential. Integration with CA125, other molecular biomarkers, and data-driven approaches may provide additional value, but improvements in sensitivity must be balanced against the stringent specificity required for OC screening and the consequences of false-positive results. Ultimately, clinical translation will require methylation assays to demonstrate not only diagnostic accuracy but also meaningful benefit within complete early-detection pathways, including acceptable downstream investigation, cost, scalability, and patient outcomes.

## Data Availability

Data sharing is not applicable to this article as no new data were created or analysed in this study.
